# Development, integration, retention, and career progression of physician associates/assistants in UK NHS hospitals and clinical teams: a multiple-case qualitative study

**DOI:** 10.1186/s12916-026-04880-2

**Published:** 2026-04-20

**Authors:** Yingxi Zhao, Rhys Swainston, Tricia Tooman, Kim A. Walker, Reece Williams, Gerry McGivern, Geoff Wong, Mike English, Attakrit Leckcivilize, Shobhana Nagraj, The ROADMAP-PA collaborator group, Yingxi Zhao, Rhys Swainston, Tricia Tooman, Kim A. Walker, Reece Williams, Gerry McGivern, Geoff Wong, Mike English, Attakrit Leckcivilize, Shobhana Nagraj, Emyr Yosef Bakker, Kate Sophia Bascombe, Mikaela Carey, Silothabo Dliso, James Thorburn Gray, Kate Harrison, Elaine Hill, Ming Kei Leo Li, Chandran Louis, Jane Frances Rutt-Howard, Jeannie Watkins

**Affiliations:** 1 Nuffield Department of Medicine Centre for Global Health Research, University of Oxford, S Parks Rd, Oxford, OX1 3SY UK; 2 Centre for Healthcare Education Research and Innovation, University of Aberdeen, Aberdeen, UK; 3 Norfolk and Norwich University Hospitals NHS Foundation Trust, Norwich, UK; 4 King’s Business School, King’s College London, London, UK; 5 Nuffield Department of Primary Care Health Sciences, University of Oxford, Oxford, UK; 6 KEMRI-Wellcome Trust Research Programme, Nairobi, Kenya; 7 Department of Public Health and Primary Care, University of Cambridge, Cambridge, UK; 8 East London NHS Foundation Trust, London, UK

**Keywords:** Task sharing, Skill mix, Extended roles, Professional boundary, Interprofessional teams, Governance, Evaluation

## Abstract

**Background:**

The NHS has introduced a range of new and extended roles in recent decades. Physician associates / assistants (PAs) have become one of the most politically scrutinised of these roles. In 2024, the UK government-commissioned Leng Review highlighted national concerns around the clarity, governance, supervision, and career development of PAs, but offered limited guidance for NHS organisations to operationalise them. This study, initiated prior to and independent of the Leng Review, examined how NHS hospitals and clinical teams in England and Scotland develop, integrate, retain, and support PAs in practice.

**Methods:**

We conducted a multiple-case qualitative study across five NHS organisations in England and Scotland. Semi-structured interviews (n = 126) and one focus group (n = 8) were undertaken with PAs, consultants, resident doctors, other team members, senior organisational leaders, and stakeholders involved in workforce planning, supervision, and governance. Data were analysed thematically using a framework informed by prior scoping review and organised across macro (system), meso (organisational), and micro (individual/team) levels, with within- and cross-case comparisons.

**Results:**

We identified 12 themes across macro, meso, and micro levels of the health system. At the macro-system level, labour market dynamics, fluctuating policy and regulatory signals, and wider public and professional debates, influenced organisational confidence in the PA role. At the meso-organisational level, PA role implementation was sometimes driven by well-intentioned local leaders responding to service needs but with short-term business case logics and pragmatic pressures, rather than long-term workforce planning. Governance arrangements were often developed retrospectively and inconsistently communicated. At the micro-team level, PAs’ interpersonal skills and contribution to continuity of care were widely valued, though progression remained highly variable and reliant on local supervision and individual negotiation.

**Conclusions:**

The development, integration, retention, and career progression of PAs in hospitals are shaped by interacting system, organisational, team and individual influences. Many of these challenges reflected wider NHS workforce implementation dynamics, rather than features unique to PAs. To continue to support safe, effective, and sustainable use of PAs, and other new and extended roles, organisations and clinical teams need strong workforce planning, local change management processes, and fair career pathways.

**Supplementary Information:**

The online version contains supplementary material available at 10.1186/s12916-026-04880-2.

## Background

Persistent staffing crises and evolving models of care have driven the introduction of new and extended roles within the NHS. These have included assistant practitioners [1], clinical pharmacists in general practice [2], nursing associates [3], and care coordinators and social prescribing link workers in primary care [4, 5]. The recent NHS “Fit for the Future: 10 Year Health Plan for England” in 2025 emphasised the need to “move beyond traditional professional boundaries”, which may constrain the performance of specific tasks and make better use of new and extended roles in a safe and productive way [6]. There has been a similar strategic commitment to new role development and redesign in other nations such as Scotland [7] and Wales [8]. However, implementing new roles in practice has often proven challenging.

Physician associates (PAs)—referred to as physician assistants in earlier UK policy and recently recommended to revert to this terminology [9]—represent a small and relatively new NHS role. Modelled on US physician assistants, UK PAs are graduate-trained in the medical educational model to assess, diagnose, and treat patients under doctors’ supervision. PAs were introduced with the aim of increasing clinical capacity and addressing workforce shortages [10]. Their proposed expansion in the NHS Long Term Workforce plan attracted significant political scrutiny, linked to media attention, around patient safety, efficacy [11, 12] and quality of care [13]. Previous empirical studies, particularly Drennan et al.’s multiple case studies of PAs in English hospitals in 2016–2017 [14, 15], have offered rich insights on what PAs do, and how they contribute to patient care in hospitals. More broadly, existing reviews indicate that PAs could practise safely and effectively when working under direct medical supervision and in post-diagnostic care [13]. However, concerns persist regarding the limited evidence base on their overall value and contribution [11]. Moreover, recent developments, including PAs’ statutory regulation by the General Medical Council in 2024, and wider policy and fierce professional discussion around the role, have further intensified scrutiny and uncertainty regarding the PA profession.

Contemporary debates about the PA role resulted in commissioning of the Leng Review by the UK government in 2024 [9]. This Review was published during the course of our study (which commenced in 2023 prior to its commissioning and independently of it) and contributed to wider policy discussions on PAs, situating the study within a critical period. The Leng Review has since drawn attention to key issues relating to PAs (and anaesthesia associates), including role clarity, supervision and training, governance, and career development, as well as variations in local implementation of the PA role [9]. While the Review made recommendations spanning national policy, regulation, and professional governance, it provided limited guidance on how these should be operationalised in NHS organisations. As of February 2026, many of the recommendations have not yet been implemented and remain subject to further consultations [16]. Our interpretation of the study findings was therefore considered in light of the Leng Review recommendations.

Currently, over half of PAs practise in secondary care settings [9]. The Leng Review recommendations stated that newly qualified PAs should gain at least two years of post-qualification experience in hospitals. These recommendations provided important policy guidance for local NHS leaders and managers as they navigate the uncertainties around the PA profession. However, there remains a lack of empirical work investigating the multi-layered organisational dynamics [17] surrounding integration of PAs and similar roles within clinical teams and hospitals, or how supervision is defined and enacted in practice across organisations and specialties [18].

Nearly a decade after Drennan and colleagues’ studies [14, 15], and within a highly politically sensitive context, we need to revisit how PA roles have evolved over time, including the long-term integration and career development of earlier entrants to the PA profession. In particular, it is important to examine how organisational and team-level processes interact, and to learn from variations across clinical teams, specialties, and organisations, including settings in and beyond England, such as Scotland.

### Aims and research question

This comparative multiple-case study builds on our earlier scoping [19] and realist review [20] by empirically examining how national, organisational, team, and individual-level factors shape the development, integration, retention, and career development of PA roles in clinical teams in NHS hospitals. This study aims to explain the successes and challenges involved in implementing and sustaining the PA role in practice.

## Methods

### Study design

This study is part of a larger National Institute for Health and Care Research (NIHR)-funded project (NIHR153324: 05/23—10/26) examining the role and adoption of PAs in NHS secondary care in the UK. This component was a comparative multiple-case qualitative study [21], drawing on embedded organisational and clinical team cases and within- and cross-case analysis to generate transferable insights. Data collection and analysis were guided by a multiple-case study logic [21], focusing on achieving sufficient depth and variation across organisational context to supports within-case and cross-case analysis.

The study was conducted across five NHS organisation: four NHS Trust hospitals in England and one Scottish Health Board hospital, studying ten clinical teams. We primarily focused on understanding how NHS hospitals and clinical teams approached the development, integration, retention, and career progression of PAs, using semi-structured interviews and focus group discussions.

Ethical approval was obtained from the Health Research Authority (Research Ethics Committee Reference: 24/NW/0116) and governance approval was obtained from the Research and Development Offices in each Trust and Health Board. This study was reported in accordance with the consolidated criteria for reporting qualitative research (COREQ) guidance (see Additional File 1).

### Setting

We purposively sampled NHS organisations to capture variations in geographic location, socio-economic catchment, the maturity of PA recruitment and deployment, and links to PA training programmes. We approached eight organisations through research and professional networks, and five were ultimately included due to feasibility considerations and their ability to support the required data collection within the study timeframe. Within each organisation, we selected two clinical teams as embedded case units (nested units of analysis within each organisational case), representing different specialties (acute and emergency medicine, general and specialist medicine, and general and specialist surgery), and varying stages of PA role development. While we initially sought to identify teams with successful and challenging experiences of PA role implementation, in practice these distinctions were often less clear-cut, with teams demonstrating strengths in some aspects of role development but challenges in others. Selection was guided by local collaborators to capture a range of contexts, including teams where PAs were well established, recently introduced, or where the role had not yet been formally implemented. For example, in Clinical Team B of Hospital B, PA students had been placed but no permanent posts for PAs had been developed, offering an example of where the PA role had not become established.

Table 1 summarises the organisational and clinical team characteristics, including organisational size, PA-to-doctor and PA-to-consultant ratios and the presence of other advanced practice roles within the two teams.

**Table 1:** Organisation and clinical team characteristics

**Organisation**	**Hospital A**		**Hospital B**		**Hospital C**		**Hospital D**		**Hospital E**	
**Organisational-level**										
Number of beds	1,000–1,500		500–1,000		1,000–1,500		500–1,000		1,000–1,500	
Number of employees	10,000–15,000		5,000–10,000		10,000–15,000		15,000–20,000		10,000–15,000	
Linkage to PA training programme (e.g. student placement)	Weak		Strong		Strong		Strong		Strong	
Start of PA within the organisation	2014		2018		2018		2013		2008	
**Team**	**Team A**	**Team B**	**Team A**	**Team B**	**Team A**	**Team B**	**Team A**	**Team B**	**Team A**	**Team B**
**Clinical team -level**										
Specialty	Acute medical services	Specialist surgical services	Specialist medical services	Specialist medical services	Acute medical services	Specialist medical services	Acute medical services	General surgical services	Specialist medical services	Specialist surgical services
PA-to-doctor ratio	1:8	1:8	1:9	N/A (No PA)	1:17	1:14	1:6	1:16	1:12	1:6
PA-to-consultant ratio	1:4	1:3	1:4	N/A (No PA)	1:6	1:5	2:3	1:5	1:7	1:2
Other advanced practice roles within the team	Yes	Yes	Yes	No	Yes	Yes	Yes	Yes	Yes	Yes
Start of PA within the team	2018	2015	2018	N/A (No PA)	2018	2018	2013	2013	2014	2016

Organisational and team characteristics were obtained from interviews, information provided by local collaborators or publicly available information. PA-to-doctor and PA-to-consultant ratios are approximate estimates based on locally available workforce data and participants’accounts. Ranges are used where necessary to preserve anonymity. Other advanced practice roles included but were not limited to advanced clinical practitioners, surgical care practitioners, advanced allied health professionals

### Recruitment

We used a combination of purposive and snowballing sampling. The research team approached participants directly through email or were introduced via local collaborators. Across all sites, we approached over 300 individuals, of whom approximately 40% agreed to participate in our study. One participant later withdrew their interview data citing concerns around the political sensitivity surrounding PA roles at the time of data collection.

We included individuals with organisational, clinical, educational responsibilities related to PAs, as well as those with broader workforce responsibilities. These included (1) senior organisational leaders, such as chief executives, chief medical, nursing, and people officers, divisional and associate directors; (2) clinical team members from the two selected teams, including PAs, consultants, resident doctors, nurses, and other allied health professionals; (3) other stakeholders involved in or influencing PA workforce discussions, including lead PAs, workforce or education managers. Organisational leaders and stakeholders were recruited purposively, whereas recruitment within the clinical teams primarily followed snowball sampling, beginning with clinical directors or PAs, who then identified additional relevant team members. In addition to individual interviews, a separate focus group was conducted with PAs in one organisation. This focus group explored shared and differing experiences on integration and career development across various clinical teams, which enabled participants to reflect collectively on the organisational and professional dynamics shaping their roles.

### Data collection

We conducted face-to-face semi-structured interviews and one focus group discussion in the hospital sites or via Microsoft Teams between Oct 2024 and Nov 2025 mostly by four experienced health and social science researchers (YZ, RS, TT, and AL [three male and one female]), none of whom are healthcare professionals and three of which are postdoctoral researchers. A PA researcher (RW) contributed to interviews with PA participants in some sites to support rapport and trust, given the sensitive political climate.

The interview guide was developed by the research team, informed by findings from our scoping [19] and realist reviews [20], and iteratively refined throughout data collection (see Additional File 2). It was flexibly adapted for different participant groups and covered topics, including organisational context and history, experiences working with or as PAs, team dynamics and relationships, and views on PA retention and career development. Interviews lasted between 30–120 min. Data collected through the interviews and focus group discussions were audio-recorded with consent, transcribed verbatim by an accredited transcription service, and anonymised for analysis. Data collection coincided with the commissioning and publication of the Leng Review [9]. We therefore also monitored relevant public statements and policy documents such as from medical royal colleges and the British Medical Association (BMA), which informed iterative adaptation of interview questions and interpretation of emerging findings.

Alongside interviews, we collected contextual information about each organisation and clinical team, including the number of PAs, relative PA-to-doctor and PA-to-consultant ratios, and the presence of other advanced practice roles (see Table 1), to guide our data collection and analysis.

### Data analysis

Data were first coded deductively using the framework derived from the scoping reviews (38 categories) [19] which provided a broad conceptual structure for examining workforce roles across system, organisational, and team levels. This deductive framework was supplemented with inductive coding to capture concepts emerging from the data that were not fully reflected in the original framework. Codes were organised into themes across macro (system), meso (organisation and department), and micro (individual and team) levels. We also conducted within-case and cross-case comparisons to examine similarities and differences across organisations and teams to identify commonalities, contrasts, and contextual influences (see Additional File 3).

YZ and RS undertook the initial coding using Nvivo (version 1.5.1), and YZ reviewed all coding and developed the thematic structures. The core research team met regularly to examine emerging interpretations, discuss alternative explanations, and refine the thematic framework.

### Reflexivity

Our research team comprised insider and outsider perspectives, fostering a collaborative approach to data collection and analysis, which enhanced contextual sensitivity, reflexivity and credibility, and aimed to reduce bias [22]. Reflexivity discussions, collective sensemaking workshops, and memo-writing were used throughout to surface assumptions and support methodological transparency. Four health and social science researchers (YZ, RS, TT, AL) and one PA researcher (RW) conducted data collection, supported by a clinician-researcher (SN). Involving a PA researcher was important for facilitating trust and open discussion among PA participants in some sites. Emerging findings were discussed with local collaborators from each organisation, involving senior clinicians, PAs, and nurses, and within a wider collaborator network as part of the broader project’s collective sensemaking workshops. This network included clinicians, PA educators, regulators, academics, and policymakers, enabling diverse perspectives to inform interpretation. A patient and public involvement group contributed early feedback on data collection and informed interpretation, particularly regarding issues related to patient understanding and perceptions of PA roles.

## Results

### Participants

We conducted semi-structured interviews with 126 participants, and one focus group with 8 additional participants, bringing the total to 134 individuals. These included 37 senior organisational leaders (central and divisional managers), 69 clinical team members (consultants, nurses, resident doctors, PAs, and other allied health professionals), and 28 other stakeholders involved in PA governance, supervision, or deployment. In total, 31 of the participants were PAs. Representation distributed across all five study sites and organisational levels is shown in Table 2.

**Table 2:** Participant distribution by organisation and clinical team

**Organisation (Confidential Code)**	**Central management** (CM)	**Divisional management** (DM)	**Team A** (TA)	**Team B** (TB)	**Other** (OT)	**Out of which**: **PAs**
Hospital A (HA)	4	3	7	11	6	3
Hospital B (HB)	9	4	7	4	4	1
Hospital C (HC)	5	3	11	4	12	13
Hospital D (HD)	3	3	8	4	4	7
Hospital E (HE)	2	1	6	7	2	7
Total	23	14	69		28	31

Participant identifiers use the format “Hospital – Group – Number (e.g. HA CM 01)”

*Abbreviations*: *CM* Central management (board or chief executive level), *DM* Divisional management (divisional or larger directorate level), *TA* clinical team A, *TB* clinical team B, *OT* Other (such as PAs working elsewhere in the organisation, outside the two focal clinical teams, and other key informants involved in PA-related governance, workforce, or education activities)

Table 3 summarises the 12 key themes that operate across macro (system), meso (organisational and departmental), and micro (individual and team) levels, with highlights of cross-case patterns and key variations. Across the ten embedded clinical teams, variation was strongly associated with workforce configurations (including PA-to-doctor and PA-to-consultant ratios), clinical specialty (acute, procedural or ward-based care), and the local history of PA deployment as shown in Table 1, which together shaped how PA roles were developed, integrated, and sustained over time.

**Table 3:** Themes across macro (system), meso (organisation and department), and micro (team and individual) levels

Level	Themes	Cross-case patterns	Key variations between cases
**Macro (health system)**	1. Workforce supply and labour market dynamics create fluctuating demand for PAs	• PA, medical and nursing workforce supply are shifting over time across all sites • Recruitment pressure and financial constraints were common across organisations	• Geographic recruitment challenges, e.g. high urban living costs in vs. relative rural unattractiveness, specialty attractiveness across profession • Variable links to PA training programmes (see Table 1) • Different staffing comparators, with PA benchmarked against international medical graduates, foundation year doctors, or advanced practice roles
	2. Policy direction and professional and public debates can enable or undermine confidence in the role	• National policy signals, regulatory uncertainty, and professional/public debates influenced confidence in PA roles across all sites • Inconsistent and contested policy messaging generated caution among organisational and team leaders	• Interviews conducted during ongoing policy debate (regulation and the Leng Review), shaping participants’uncertainty about regulatory direction • Differences in national policy context (England and Scotland) shaped how organisations interpreted and anticipated policy changes • Organisational responses ranged from cautious role restrictions to stronger leadership advocacy. Sites with both larger and a longer history of PA workforces showered greater leadership support
	3. Regulation and prescribing rights constrain scope and role activities, leading to inconsistent expectations	• Absence of prescribing rights was consistently raised across sites	• The practical impact of prescribing and scope of practice constraints varied by clinical setting and patient type • Teams where PAs had previously undertaken advanced procedures experienced cautious restrictions
**Meso (organisation and department)**	4. Short-term business case logic drives PA adoption rather than systematic workforce planning	• PA roles were commonly introduced using short-term business cases • Financial viability strongly shaped role approval and sustainability	• Degree of workforce planning ranged from formal process to ad hoc initiatives • Responsibility for PA planning at different levels (organisation-led or team-led) • Assumptions about PA deployment varied, including whether PAs or other advanced practice roles were prioritised, and the ‘ideal’PA-to-doctor ratios varied widely between sites (as reflected in Table 1)
	5. Leaders and champions shape the development, integration and progression of PA roles	• Clinical leaders and champions were central to initiating, legitimising, and sustaining PA roles • Leaders with direct experience of working with PAs generally held positive views of their contribution	• Strength of leadership support ranged from active champions who enabled role progression, to more cautious leaders who sought to keep PA roles aligned with their initial job descriptions and expectations • Alignment of leadership support varied across organisational layers (unit, division, and organisation) and between clinical and general managers
	6. Supportive and collaborative organisational culture enables integration and retention, while negative climate increases attrition	• Supportive and collaborative team cultures consistently facilitated PA integration and day-to-day functioning across sites, and in some cases PAs’continuity helped sustain such cultures	• External pressures such as media and policy debates affected local team climates differently across organisations • Not all units explicitly recognised organisational culture as a factor in PA retention
	7. Governance structures for PAs are often retrospective, and inconsistently managed and communicated	• Governance arrangement for PAs was developed retrospectively in many sites • Formal governance documents existed in most sites but had limited visibility in everyday practices	• The formality and maturity of governance varied, often linked to both the size and the history of the PA workforce • Induction practices differed, some teams made deliberate efforts to explain PA roles to new staff, and others treated PAs as already normalised • Symbolic markers such as uniforms varied across teams, subtly shaping how PA roles were perceived
	8. Resources shape PA development, retention, and sustainability, but scarcity makes them vulnerable	• Financial resources were a pre-requisite for PA role development across all sites • Time for supervision, induction, and ongoing support was widely recognised as essential but often constrained	• Funding models differed, with some organisations initially relying on external or pump-priming funding, while others used cost-neutral business cases • Local resource pressures for PAs differed by organisational financial position, specialty, and clinical setting
	9. Limited approaches to evaluation and uneven representation make it hard to showcase PA contributions	• While PAs were valued for continuity, evidence of service delivery impact was limited across sites • PAs reported heightened sensitivity around representation and visibility in the current political climate	• Formal evaluation, audit, or approval was uncommon, and where undertaken, findings were not widely shared or visible • PA representation varied, with some sites having designated lead PA roles at organisational-level or unit-level, while others had none or discontinued the role
**Micro** **(team and individual)**	10. Individual motivations shape PA job choices and career paths but unclear progression leaves much to individual initiative	• Individual motivations and preferences strongly shaped PA job choices across sites • Participants described a tightening PA labour market, with reduced flexibility compared with earlier cohorts • A shared perception of limited and uncertain long-term career progression	• Access to formal progression opportunities differed, with some organisations offering lead PA roles, while others had no structured routes • Career trajectories varied between PAs developing specialist or portfolio roles, and those remaining in stable, generalist posts, often depending on local supervisory support, availability of opportunities, and the size and configuration of teams (for example see Table 1 for PA-to-doctor ratios)
	11. Individual attributes, competence, and evolving supervision shape PA role activities and autonomy	• PAs were widely valued for continuity, local knowledge, and organisational expertise • Role development often occurred through evolving supervision and gradual progression in responsibility	• Approaches to supervision differed by specialty and settings, with greater concerns expressed by teams less familiar with PAs or working outside acute settings • PA role trajectories varied, shaped by individual skills and prior experience, service configuration, and negotiation with supervisors
	12. Team and patient perceptions of PAs are mixed, shaping integration but rarely influencing role or career development	• Limited understanding of why PAs were introduced was common among resident doctors, nurses and rotating staff • Perceived patient understanding of the role was generally low across sites	• Team dynamics varied with relative numbers of PAs and doctors with PAs more readily normalised in teams with higher PA presence (see Table 1) • Patient expectations differed by settings, e.g. acute care vs. specialist services

### Macro-level

Across the five study sites, wider workforce supply and changing labour market dynamics shaped both opportunities and constraints for developing and introducing PAs. Initially, growing service and educational pressure as well as shortages of doctors and nurses (particularly in hard-to-staff regions and specific specialties), were described as creating demand for alternative workforce solutions. At that time, there was also a good supply of high-quality PA graduates, which contributed to PAs being seen as a pragmatic option. In some sites, however, more recent increases in medical and nursing staffing (partially due to expanded training and international recruitment) altered local staffing priorities and, in some contexts, reduced the perceived urgency for additional PAs. As one clinician manager reflected on the labour market dynamics of their region in the context of recent increases in medical training numbers:

*We don’t have shortage of medical staff anymore, at least temporarily…there were these...F1s [newly qualified doctors] this year, who didn’t have placements in the country and we were asked to run around and find, create posts out of no money for them… So I think, at least temporarily, the PAs were at one point going to be the solution to the staffing crisis. We no longer have a staffing crisis. – HA DM 02 Clinician manager*

National policy direction and professional and public debates played a major role in shaping confidence in the PA role across all sites. Early policy signals, including endorsement from national and regional organisations and the availability of pump-priming funding, helped stimulate interest in developing and recruiting PA roles. Interviewees contrasted this earlier period of policy support and relative confidence with the more recent phase characterised by heightened scrutiny and uncertainty. The lack of sustained and coherent policy messaging, alongside highly visible and often polarised public and professional debate from government, professional bodies, and the media, created uncertainty and made organisations increasingly cautious about further investing in the role. Additionally, in the Scottish site, interviewees expressed uncertainty about how England-led policy developments, such as the Leng Review, would translate legally or operationally into the Scottish context. In contrast, advanced practice roles such as advanced clinical practitioners in many sites were sometimes described as having clearer legitimacy, established governance, and stronger external and internal support, which led some organisations to prioritise their development over PAs. Many organisational managers expressed a continued need for clearer national direction to mitigate confusion:

*I don’t know how…the Leng Review is going to turn out, how the various political campaigns are going to turn out, but I think it’s got to a very unnecessary and unpleasant stage...We need a bit more of a national steer and national guidance about how to do that safely without causing too much distress for the individuals. – HB CM 06 Clinician manager*

These negative external policy and political discourses also affected staff morale and behaviour. In some sites, PAs became hesitant to engage in visible clinical roles, such as induction or teaching, reduced certain clinical activities, and in several sites chose to reduce working hours or reconsider long-term career intentions.

*Everything going on in the news at the moment won’t be helping…The physician associates in our department, they do the induction for the junior doctors…with everything that’s been going on recently, they’ve been a bit hesitant to even do the induction in certain cases. – HD TB 02 PA*

*We do read those bits, and it does affect how when you are working…it does affect your psyche a lot... And then when you come to work, you are cautious. – HA TA 01 PA*

Uncertainty surrounding regulation, and by extension prescribing rights, further shaped role development and led to inconsistent expectations across organisations and teams. In absence of statutory regulation before 2024, limited awareness of quality assurance for PA education or relevant national guidance, and more recently highly restrictive directives issued by some professional bodies, there was limited shared understanding about PAs’ scope of practice and opportunities for further development. Across all sites, this resulted in confusion and different expectations among team members.

*There was no national consensus [about] what we [PAs] can or can’t do right at the beginning, in terms of advanced procedures... opinions varied, and I think that’s where there’s some peers that do things and some peers that actually don’t do things in different hospitals. – HC PA focus group*

In some teams, this led to conservative approaches to PA activities and role development, particularly where PAs had previously been permitted to undertake advanced clinical tasks.

### Meso-level

At the organisational level, PAs were often introduced through short-term business cases and pragmatic decisions, rather than systematic workforce planning. In several sites, managers adopted what was described as a ‘wait and learn’ approach to piloting PA roles at the departmental level. While formal business cases often referenced service needs and team continuity, participants suggested that financial considerations were central for approval by organisational leaders:

*I wrote the business case for the first in [organisation] PAs a number of years ago, supported at that time by the Director of Medical Education, because our training was in crisis, and we were also spending a lot of money on locum, short term locum, FY2s [Foundation Year 2 trainee doctors] for the ward. And, so, we put back together in a cost avoidance business case, to appoint the first in [organisation] PAs, to improve the quality of our training, improve the quality of care provision on the wards, and to reduce costs. – HA – TB 02 Clinician manager*

The emphasis on financial viability in some sites made PAs particularly vulnerable when budgets tightened or when external funding was unavailable, limiting long-term investment in their development and growth.

Development, integration, and progression of PA roles were highly dependent on local leaders and champions. Perceptions of the value and positioning of PAs varied across organisational levels, particularly between medical, nursing, and organisational leaders. Clinically engaged leaders, especially at the unit level, often acted as PA advocates and champions, creating the supportive environment for PAs to develop, grow and explore new responsibilities, as illustrated below:

*Our clinical director is also our undergrad lead as well, supports them [PAs]. And I think it really is dependent on what your clinical director feels about them, and then how you embed them into an area. And I truly believe that. – HB CM 09 Manager*

By contrast, in teams where senior clinical leaders were sceptical about PAs (or about new and extended roles more broadly – for example, as seen in Hospital B Team B), PA roles were not actively developed or prioritised. In some teams, especially those with a longer history of PA employment, leadership changes over time weakened shared understanding of the original rationale for the role. At the same time, some frontline staff felt that organisational leaders held somewhat idealised or *“utopian”* expectations of PAs that did not reflect the complexities of frontline work or interprofessional dynamics.

Supportive and collaborative organisational culture enabled PAs integration and retention. Teams with collaborative dynamics, flatter hierarchies, and strong working relationships between doctors and nurses provided conditions in which new roles could be embedded more smoothly. Such culture fostered satisfaction, created spaces for role identity formation, and supported continuity of working relationships. They also promoted psychological safety, which is an important condition for integration, by enabling team members including PAs to voice concerns, ask questions, and participate fully in the team. By contrast, in teams with rigid hierarchies or strained interprofessional relationships, PAs reported greater challenges integrating with some choosing to leave. While recent external discourses occasionally strained local climates, these were not consistently linked to retention in our data:

*As a department...there is no hierarchy and everyone gets called by their first name, and everyone gets made fun of, or joking about with consultants who are in their 50s…We’re very good at doing things as a social aspect and acting like basically a family, and anyone who voices any concerns is taken very seriously. – HD TA 08 PA*

Governance structures for PAs were often created retrospectively and inconsistently managed and communicated. In many organisations, policy documents, frameworks, and governance arrangements were introduced after PAs had been working for some time, and in several sites especially the later adopters, they were still under review. Awareness of these documents among managers, supervisors, or team members was often limited, which meant that their impact was limited and largely symbolic:

*I didn’t feel there has been significant change since [the hospital-specific governance policy], apart from there is now just a document that supports their [PA] role.… I guess what it probably has done is created some awareness for the rest of the workforce within the organisation… more understanding of what that role is and how they can work together with them. – HE DM 01 Manager*

However, this challenge was not unique to PA roles but applied to many other workforce documents. At the unit level, practical decisions, such as induction, uniform, and rota management, were handled informally and locally, leading to inconsistency in implementation.

Resource availability, particularly funding, time, and supervision, shaped PA development, retention, and sustainability. Where these resources were constrained, PA roles were vulnerable to contestation and rollback. Funding arrangements for PA positions were often unclear or externally driven, with managers often inheriting budgets and lacking clarity on their origins. Investment in PA training or progression was constrained, partly due to wider financial pressure across study sites. Training resources were also contested. While some clinical team members believed PAs relieved pressure and enhanced learning opportunities, others viewed them as potentially limiting training experiences. However, this concern was sometimes linked to uncertainties among trainees about their expected learning and what the system could offer in terms of training opportunities, rather than stemming from the presence of PAs per se:

*I haven’t attended any clinic or [specific procedure] or referrals on this rotation, but I haven’t asked for it, to be honest. But I do know that [PAs] do get those opportunities. I wouldn’t say that they’re necessarily taking them away because I haven’t asked for it. – HB TA 01 Resident doctor*

One consultant described the complexity relating to medical training and the implementation of PA roles:

*You want to develop your service so that... the registrars can...do the better or bigger or more complex or whatever stuff that maybe doesn’t come around so much and [PAs] are doing more of the humdrum stuff. But...I just don’t get it...stealing training opportunities... I’m not saying that doesn’t happen. I just think if there are trainees and they’re not getting access to do the training that they need to do to become senior trainees and consultants and because of a PA that just seems bizarre to me. – HD TA 05 Clinician*

Finally, showcasing PA impact remained challenging due to limited evidence, inconsistent representation, and concerns about fairness. Many sites reported benefits such as improved continuity, teamwork, patient communication, and at times positive experiences led to further employment of PAs. However, such insights were rarely backed by formal evaluation or local audits, reflecting the early and evolving nature of many new roles. Even when audits or appraisals were conducted locally, awareness of the findings was limited and feedback loops were often weak or absent:

*I don’t think there has been any real evaluation. The three of us who do the mentoring for each of the PAs, there was no appraisal system for them. So, I designed an appraisal that I used based on the GP appraisal…But we fill these things in and sent them to the clinical lead for the unit. There was never any feedback…No point in you doing an appraisal if nobody reads it. – HA TA 07 Clinician*

Some managers feared that evaluating a small professional group could be discriminatory and unfair. Lead PA roles helped raise PAs’ visibility, yet representation remained patchy across sites, and contemporary national debates made it harder to highlight positive contributions.

### Micro-level

At the individual level, PAs’ career trajectories reflected personal motivations, but unclear progression pathways meant advancement relied heavily on individual initiative. Across all sites, PAs who graduated earlier, during the expansion of the PA workforce, often chose posts based on their student or internship rotation experiences and specialty interests. However, recent graduates reported that they tended to apply for whatever role was available, due to the tightened labour market. Progression relied heavily on personal drive and supervisor support, with some from the early PA cohort role-modelling their development informally on medical training pathways. Some PAs pursued specialist or portfolio-style career across multiple teams, commonly in larger teams with higher PA-to-consultant ratios, while others viewed the job as a stable 9–5 job. The absence of clear advancement pathways also led some to leave the profession or re-train in medicine due to perceived career ceilings. One PA reflected on these limitations:

*I think progression wise, it’s...very limited, which didn’t really factor in when I was doing my training, but now I’m in the role...it’s become more of a focus. I think that if we are to keep the role in the UK...I think they need to look at somehow identifying a way for progression or just some form of progression, because if you top out of Band 7 [one NHS pay scale] at the age of 25, 30, there’s no further progression for you to go and do extra additional training or more senior roles. It limits your scope a little bit. – HC - TB 04 PA*

Individual attributes, competence, and evolving supervision shaped PA role activities and autonomy, though this also contributed to wide variability across teams. PAs entered with diverse backgrounds. For example, some had worked as nurses or allied health professionals, which alongside strong communication skills and enthusiasm, helped build trust and acceptance among clinical colleagues. However, these strengths were often perceived as linked to an individual’s personality rather than the PA role itself. PAs were often valued for their administrative knowledge, continuity, and expertise in local processes.

*The very thing that their communication skills are very good in terms of communicating with their colleagues, doctors, nurses. The other thing, they communicate very well with patients, their depth of knowledge when it comes to the acute medical condition is surprising, like it’s not what I expected… They’ve been amazing so far actually…they know the local policies, they know all the guidelines. – HC TA 08 Resident doctor*

Over time, supervisors adjusted levels of oversight based on PAs’ observed performance, enabling some PAs to take on more responsibility, develop autonomy, and evolve into different role configurations. While this gradual progression supported growth, it meant that PAs needed time to build their confidence. It also raised concerns among trainee doctors, particularly those rotating into teams and unfamiliar with PA training and local role arrangement, who questioned how autonomy developed in practice, in relation to differences in training pathways, regulation, and assessment structures. One resident doctor commented on the variability of PA below:

*I’ve found them highly variable, where an F1 [Foundation Year 1 trainee doctors] normally has a predictable level of practice. I’ve found that the PAs are quite different in how they operate, and it takes a little bit of time to then get used to working with them, so that you know what they’re capable of, as opposed to one of their colleagues, who I mean have the same qualification… I don’t know if the training programmes are standardised or not. – HA TA 03 Resident doctor*

Team and patient perceptions of PAs were mixed, influencing day-to-day integration, but rarely influencing role or career development. Decisions about developing and recruiting new roles were made primarily by organisational and team leaders, rather than based on direct input from clinical team members or patients. While consultants often valued PAs for continuity and reliability, some trainees, rotating doctors, and advanced practice staff reported confusion and tensions around role boundaries and responsibilities. PAs actively navigated these dynamics, often relying on peer support.

*It was challenging at first, being the first one [PA]. A lot of people hadn’t heard of the role. If they had heard of the role, they didn’t really know what I could or couldn’t do, so there was a lot of education and learning on both sides…It took a good few months, I’d say, just to find my feet, and also just work out where I can fit in. – HE TA 02 PA*

Patient experiences were shaped more by individual PA’s interpersonal qualities than by their professional titles and although some patients occasionally requested to see a (senior) doctor, this was uncommon in urgent hospital settings.

## Discussion

This multiple-case qualitative study examined how NHS hospitals and clinical teams approached the development, integration, retention, and career development of PAs. We found that the PA role trajectories were shaped by interacting factors at the macro, meso, and micro levels.

At macro-health system level, fluctuating policy and regulatory signals, and wider professional and public debates strongly influenced organisational interest and confidence in the role. At the meso-organisational level, PA role implementation was often driven by well-intentioned local leaders and champions, as well as through short-term business case logic and pragmatic pressures rather than long-term systematic workforce planning. Governance arrangements were sometimes developed retrospectively and were inconsistently managed and communicated. At the micro-individual and team-level, the PA role was significantly shaped by individual PA’s attributes, local supervisory arrangements, and team culture. PA’s interpersonal skills and contribution to continuity of care were not only valued but also influenced how well they were perceived and accepted by team members. We also found that although leaders and team members’ perceptions of patient views and team dynamics influenced day-to-day integration, these perspectives did not necessarily impact longer-term workforce decisions, which were largely determined by organisational and clinical team leaders.

Our previous scoping [19] and realist reviews [20] have identified a range of contextual and organisational factors that supported or impeded PA role development. This study extends those findings by demonstrating how organisations make difficult workforce and service delivery decisions over time. We show that PA role adoption was often shaped by short-term operational pressures and service needs, including efforts to reduce reliance on locum staffing [23], rather than by long-term strategic workforce planning. In some sites, participants indicated PAs were introduced primarily following the availability of external or pump-priming funding availability, rather than through deliberate workforce planning. Like evaluations of new roles in mental health trusts [24], and the additional role reimbursement scheme in primary care [25], we found that a ‘piecemeal’ workforce logic triggered by short-term funding opportunities drove role development in some organisations, with managers using the funding based on availability [25]. This reflects wider concerns about the unintended consequences of short-term policy interventions [26], which can limit attention to governance, and long-term workforce and career planning, resulting in roles that were variably embedded, vulnerable to disruption, and sometimes deployed in ways that diverged from their original intent.

A further contribution of this study shows how institutional memory was often weak, particularly around how decisions were made, how posts were funded, or what governance arrangements existed for PAs, especially following leadership change and passage of time [27]. Many clinical team-leads, even in teams with a long history employing of PAs, lacked clarity and a shared understanding on the original rationale for introducing the role, which led to uncertainty about its positioning and justification. Consequently, the PA role was vulnerable to disruption and opportunities for more advanced procedures could be withdrawn, especially when new trainees arrived or when external debates triggered caution or resistance. This highlights that even once established, new roles are not necessarily secure; their integration is not linear but rather dynamic, negotiated and potentially reversible [28], and shaped by local dynamics, policy signals, and shifting professional discourse.

Our findings are supported by Drennan and colleagues’ earlier research on PAs [14], and builds these themes further by providing a deeper examination of how PA roles are managed and interpreted at both organisational and clinical team levels. We show that, as is common with new and extended roles, many core decisions about role scope, supervision, and progression were made locally and at the discretion of the clinical team leads, rather than being directed by senior hospital leaders [17]. This contributed to significant intra-organisation variation in how PA roles were deployed, governed and progressed, highlighting the need for deliberate role planning and governance at the local team and departmental level.

Across the five hospitals and ten clinical teams, we observed broadly similar challenges relating to national policy uncertainty and current financial pressures leading to stalled role development. However, our comparative analysis also revealed important variations in how PA roles were understood, adopted, and operationalised in each case. Comparing English and Scottish sites highlighted how national policy signals shaped interpretation differently. Interviewees in Scotland expressed uncertainty about how England-led developments, such as the Leng Review and statements from some medical royal colleges, would translate legally and operationally into the Scottish context.

The proximity to PA training programmes also played a noticeable role in shaping organisational process between cases. In some organisations, particularly Hospitals B and E, where links to local PA training programmes were particularly strong, a continuous pipeline of graduates created both opportunities and urgency to recruit PAs. However, this sometimes led to adoption that was driven by availability, rather than strategic workforce planning, reinforcing a reactive rather than intentional logic [25].

Additionally, workforce configuration and task allocation varied significantly between different cases, even within similar specialties. In some teams, PAs supported generalist ward-based care, while in others they contributed to specialist procedural or outpatient clinic roles. These patterns map closely to previously described models of skill-mix changes by Nancarrow and Borthwick [29] and others [5]. These include diversification or innovations (e.g. when PAs are taking on new and neglected activities such as tracking and following up patients discharged from one acute ambulatory unit, work that was clinically important but not clearly owned by any professional group), specialisation (e.g. when a PA developed skills in a specific procedure), or substitution/reallocation (e.g. when PAs undertook routine medical tasks that would traditionally be done by resident doctors, such as clerking and documentation). While partially driven by local service needs and departmental workforce size (for example the PA-to-doctor and PA-to-consultant ratios as shown in Table 1), the tasks are strongly dependent on the background and skills of individual PAs, and were often subject to constant negotiation, echoing recent studies of skill-mix changes in primary care [30, 31].

Lastly, through cross-case comparison, we found that clinical team leads and members tended to view PAs’ supervision in *their own setting* as appropriate, while expressing stronger concerns about supervision *in settings they were less familiar with*. Clinical team leaders and members with little or no direct experience working with PAs were most cautious. Across all sites, many participants voiced anxiety about supervision in primary care. Similarly, participants from specialist medicine and surgical teams with PAs that we studied raised concerns about supervision in acute settings, whereas those in the acute teams we studied (Team A in Hospitals C and D) generally reported that supervision was appropriate in practice.

### Policy implications

Our findings, generated independently and prior to the publication of the Leng Review, reinforce key observations also identified by the Leng Review [9] and the King’s Fund [32], particularly the consequence of unclear national vision, fragmented governance, and insufficient workforce planning. Many new and extended roles have been introduced to the NHS since it was established, and more will continue to be needed as healthcare becomes more complex. For example, a recent study showed 80 new roles in mental health trusts alone [24]. Based on our findings, we propose four key policy recommendations for organisational leaders and managers when developing and managing new and extended roles.

First, clear strategic purpose for the role is needed. These must be articulated, shared, and sustained over time across organisational and team layers, not only within teams that currently employ PAs. While PA roles may be initiated by specific clinical teams in response to local service needs, Trusts and Health Boards have a coordinating responsibility to align team-level implementation with organisation-wide workforce planning, particularly where national direction is evolving. This is especially important given the recommendation for newly qualified PAs to complete two years in hospital settings [9].

Second, local change management needs to extend beyond symbolic governance documents. They need to ensure that line managers, clinical supervisors, and team members have the capacity, knowledge, and support to operationalise these role expectations in day-to-day practices. Strengthening visibility of educational governance including national curriculum and assessment standards and links with education providers is also important, as limited awareness in some teams contributed to uncertainty about supervision and capability. Organisational leaders at Trusts and Health Boards have a role in actively supporting and coordinating local implementation, including clarity around supervision arrangements, lines of responsibility, and mechanisms to preserve shared understanding and role expectations as teams and leaders change. Without such support, governance remains largely symbolic.

Third, there is need to develop structured and equitable career and progression pathways. One example as recommended in the Leng Review [9] was to establish advanced PA roles, rather than rely on individually negotiated arrangements that risk inconsistency and limit sustained development. This includes the operationalisation of upcoming national work on career development frameworks [33], with transparent expectations around development opportunities and progression milestones, and the potential to take on additional responsibilities commensurate with training and experience (including prescribing rights, as recommended by the Leng Review [9]). These need to be agreed at the organisational level while retaining flexibility to respond to local service needs, to reduce inconsistency between teams, and support sustained progression and retention.

Finally, evaluation of new roles should examine how they evolve in practice. It should also consider the conditions that enable them to be implemented and sustained safely and effectively over time, particularly within multi-professional teams. This requires moving beyond narrow expectations for randomised experimental designs alone. Workforce roles are context-dependent and evolve through interaction with other professions over time, randomised trials often fail to capture how roles are implemented, adapted, or sustained. At the same time, over-reliance on learning from short-term pilot projects can also encourage ‘pilot-itis’ [34], producing fragmented insights that fails to inform long-term planning, scale-up, or redesign.

### Strengths and limitations

This study offers rich insights by drawing on in-depth qualitative work in five NHS organisations and ten embedded clinical teams. The multi-site, comparative design allowed for both within-case and cross-case analysis, capturing variation across specialties, organisations, and policy contexts. However, our study also has limitations, which highlight opportunities for further research.

While we initially aimed to identify contrasting ‘successful’ and ‘unsuccessful’ clinical teams, this proved difficult in practice, as the success or failure of PA role implementation was rarely clear-cut and often changed over time. In some cases, institutional memory was limited, making it challenging to study teams in which the role had been attempted but subsequently abandoned. We were only able to include one team where PA students had been and are still being placed but no permanent posts had been created. However, we believe that our study reached sufficient explanatory power [35] through the depth of data, heterogeneity of participants, and the comparative design.

Our purposive and snowball sampling strategy may have led to an under-representation of individuals with strong reservations or negative experiences of working with PAs. We sought to mitigate this by including participants such as resident doctors in other clinical teams and those known to hold more critical views in broader debates questioning PA roles. Their inclusion allowed us to capture diverse perspectives and avoid overly positive accounts. Nonetheless, there remains a risk of recruiting participants with similar views, which may have limited the diversity of perspectives captured. Additionally, only one focus group with PAs was conducted due to small numbers at some sites and practical constraints. However, findings from this group were consistent with the interview data.

Finally, this study was conducted during a period of controversy and policy development, which influenced some individuals’ willingness to participate and influenced how they narrated their experiences. One PA withdrew after the interview citing emotional and political concerns. To build trust and support open discussion, a PA researcher was added to the data collection team for some sites, and the interview guide was iteratively adapted to reflect the changing context.

## Conclusions

In this multiple-case qualitative study of five NHS organisations and ten embedded clinical teams, we show that the development, integration, retention, and career development of PAs are shaped by interacting macro, meso, and micro-level influences.

At the macro-level, national policy and regulatory signals and public and professional debates have influenced organisational interest and confidence in the PA role. At the meso organisational and departmental level, the PA role has been frequently driven by short-term business case logic and pragmatic pressures, rather than systematic workforce planning. Weak institutional memory, retrospective governance, and highly variable local implementation of the role have left many PA roles inconsistently embedded and vulnerable to contestation and rollback. At the micro-level, individual PA attributes, supervisory arrangement, and team culture shaped how roles evolved in practice.

Many of these challenges reflected wider NHS workforce implementation dynamics affecting new and extended roles more broadly, rather than features intrinsic to PAs. As the NHS continues to introduce new and extended roles in response to changing service needs, there is a pressing need for clear strategic planning, strong local change management process, and fair and transparent career pathways, to continue to support safe, effective, and sustainable role integration.

## Supplementary Information

Additional file 1. COnsolidated criteria for REporting Qualitative research Checklist.

Additional file 2. Interview guide.

Additional file 3. Thematic analysis coding table.

## Data Availability

The data are not publicly available due to privacy or ethical restrictions.

## Abbreviations

BMA: British Medical Association
COREQ: COnsolidated criteria for REporting Qualitative research
NHS: National Health Service
NIHR: National Institute for Health and Care Research
PA: Physician associate/assistant
