# Supplementary material for: Development, integration, retention, and career progression of physician associates/assistants in UK NHS hospitals and clinical teams: a multiple-case qualitative study
Source: BMC Med. 2026 Apr 20;24:276. doi: 10.1186/s12916-026-04880-2 (PMC13130418; doi:10.1186/s12916-026-04880-2)
Supplement: Supplementary file 2 — Additional file 2. Interview guide. [file 12916_2026_4880_MOESM2_ESM.docx]

***Semi-structured interview and focus group guide for physician associates***

| Question |
| --- |
| *PA background and characteristics* |
| 1. What is your background? How did you choose to be a physician associate? |
| 1. What does being a PA mean to you? |
| 1. How long have you been practising at this hospital and this team? Have you worked anywhere else as a PA? |
| *PA development and recruitment* |
| 1. Tell me about when you first started at this hospital in your role? |
| *PA role and integration* |
| 1. Please tell us about your current role in this hospital and clinical unit? |
| 1. What are the key benefits and challenges or negative experiences of working as a PA here? |
| 1. Do you work with a supervisor clinician? How would you describe that relationship? |
| 1. What’s your interaction with other health care professionals like? |
| 1. How would you describe similarities and differences between your role and others like FY, SAS and LED doctors, nurse practitioners? |
| 1. How do you describe your role to patients? |
| 1. What would success in your role look like? For example how would you describe a perfect working environment? How would you feel best supported? *[IF TIME PERMITS]* |
| 1. Have you heard much about the regulation of PA? If so, what’s your understanding of what’s happening? Do you think your role would change with regulation/what would change if you were regulated? |
| 1. How has the discussion of the PA role both within the healthcare community and the mainstream media over the last year affected you? |
| *PA retention and career progression* |
| 1. Tell me about your experience of professional development support here? |
| 1. What’s your future career plan? |
| *Closing* |
| 1. What advice or feedback would you give to a PA student or graduate looking to work in your setting/a similar setting to yours? *[IF TIME PERMITS]* |
| 1. Any other additional comments or information you’d like to share? Are there any questions that you feel were missed during this interview that are relevant to your role? |

***Semi-structured interview and focus group guide for managers***

*(This will also include the unit manager level and above)*

| Question |
| --- |
| *Manager background and characteristics* |
| 1. Could you tell me a bit about your role in this organisation? How long have you worked in the NHS? At this location? |
| 1. How did you first hear about PAs? What did you think the role of PA is? |
| *PA development and recruitment* |
| 1. How long has your hospital or department employed a PA? |
| 1. Did you have any previous experience working with or employing a PA, before working at this organisation? *[IF TIME PERMITS]* |
| 1. Were you the person who made the decision to integrate a PA into your department/division/hospital? What prompted the decision? |
| 1. What are the key challenges recruiting PAs in this department/division/hospital? And how big are those challenges? |
| *PA role and integration* |
| 1. How many PAs do you employ/manage in your department/division/hospital? |
| 1. Can you describe to me the role of PA in your department/division/hospital? |
| 1. What are the differences between the roles of PAs and others like FY, SAS and LED doctors, AA, SCP, ANP? |
| 1. What are the impacts or positive changes PAs bring to this department/division/hospital? |
| 1. What are the barriers that impact the PA role? What are the challenges of managing a PA in this setting? |
| 1. How do junior doctors and medical students in your department/division/hospital feel about PA roles? |
| 1. How do other advanced nurse practitioners in your department/division/hospital feel about PA roles? *[IF TIME PERMITS]* |
| 1. How do patients/community feel about being seen by a PA? |
| 1. How are PA roles funded in this department/division/hospital? |
| 1. How is indemnity covered for PAs in your hospital/ division/department? |
| 1. Have you heard much about the regulation of PA? If so, what’s your understanding of what’s happening? Do you think their role would change with regulation/what would change if they were regulated? *[IF TIME PERMITS]* |
| 1. How has discussion of the PA role both within the healthcare community and the mainstream media over the last year affected them? *[IF TIME PERMITS]* |
| *PA retention and career progression* |
| 1. What’s the turnover rate and retention rate of PAs and other staff like in this organisation? Can you tell me why? |
| 1. What are the available resources for PA’s professional development here? |
| *Closing* |
| 1. What advice would you give a colleague, or another organisation interested in hiring a PA? *[IF TIME PERMITS]* |
| 1. Any other additional comments or information you’d like to share? Are there any questions that you feel were missed during this interview that are relevant? |

***Semi-structured interview and focus group guide for supervising clinicians and other members in a clinical team***

| Question | Probe |
| --- | --- |
| *Team member background and characteristics* |  |
| 1. Could you tell me a bit about your role in this organisation? How long have you worked in the NHS? At this location? |  |
| 1. How did you first hear about PAs? What did you think the role of PA is? |  |
| *PA development and recruitment* |  |
| 1. How long have you worked with a PA? |  |
| 1. Did you have any previous experience working with a PA, before working in this team? | - Any similar experience? |
| 1. Were you involved in the decision to recruit and integrate a PA into this team? Tell me about the story? | - What prompted the decision? Decision at which level? - Can you describe any resistance or support in developing this role? - Why PA was chosen instead of other roles like AA, SCP or ANP? (apply to doctors) |
| *PA role and integration* |  |
| 1. How many PAs do you supervise/work within your team? |  |
| 1. Can you describe the role of the PA/PAs in your team? | - What are their key tasks or responsibilities? - Have this evolved over time? - Is PA part of a multidisciplinary team? - Emergency out of hour working? Work hours? |
| 1. What kind of PA role would you think the team would benefit from? What would be a ‘successful’ PA role look like to you? |  |
| 1. What are the differences between the roles of PAs and others like FY, SAS and LED doctors, AA, SCP, ANP? | - Differences in roles, tasks and responsibilities, knowledges - Any ‘technical’ authority/expertise that exclude PAs from doctors? - What is the unique value of PA that others cannot provide? |
| 1. How has having PAs working here impacted the team? Can you give any examples? | - Any specific examples of positive impact? - Are there any data supporting this? - Relationship with other team members? - Has there been any ‘failed’ or ‘unsuccessful’ cases? |
| 1. How has supervising/working with a PA impacted you personally? | - What about indemnity cover? (for doctors only) - Decision at which level? |
| 1. How do junior doctors and medical students in your team feel about PA roles? | - What’s their understanding? - How is PA introduced and integrated? |
| 1. How do patients/community feel about being seen by a PA? | - What do they know about PA? |
| 1. Have you heard much about the regulation of PA? If so, what’s your understanding of what’s happening? Do you think their role would change with regulation/what would change if they were regulated? |  |
| 1. How has discussion of the PA role both within the healthcare community and the mainstream media over the last year affected your perception of PAs? | - What about the strikes in 2023? |
| *PA retention and career progression* |  |
| 1. What’s the turnover rate and retention rate of PAs and other staff like in this team? Can you tell me why? |  |
| 1. What do you think a PA needs in terms of CPD and career development? | - What’s your role of providing those? What are your expectations? |
| *Closing* |  |
| 1. What advice would you give a colleague, or another department interested in hiring a PA? *[IF TIME PERMITS]* |  |
| 1. Any other additional comments or information you’d like to share? Are there any questions that you feel were missed during this interview that are relevant? |  |
