# Supplementary material for: Development, integration, retention, and career progression of physician associates/assistants in UK NHS hospitals and clinical teams: a multiple-case qualitative study
Source: BMC Med. 2026 Apr 20;24:276. doi: 10.1186/s12916-026-04880-2 (PMC13130418; doi:10.1186/s12916-026-04880-2)
Supplement: Supplementary file 3 — Additional file 3. Thematic analysis coding table. [file 12916_2026_4880_MOESM3_ESM.docx]

Additional file 2. Thematic analysis

***How NHS hospitals and clinical teams approach the development, integration, retention, and career development of physician assistants / associates: a multi-site qualitative study in England and Scotland***

| **Category** | **Main findings** | **Supportive quotes** | **Similarities between sites and units** | **Differences between sites and units** |
| --- | --- | --- | --- | --- |
| **Recruitment or development** | | | | |
| **Macro – APP and other workforce supply** | - PA roles were initially developed and recruited primarily due to challenges in hiring doctors and nurses. Compared to medical and nursing roles, there were more PA applicants and also of good quality, making them an attractive staffing solution - However, labour market dynamics have shifted, with more doctors and nurses available now, and even cases where resident doctors being unemployed. The original staffing crisis has eased to some extent, reducing the perceived need for additional PAs | **Well, the other thing of course is the fact that we don't have shortage of medical staff anymore, at least temporarily. So as I'm sure you're aware, there were these or fine F1s this year who didn't have placements in the country and we were asked to run around and find, create posts out of no money for them.… So I think at least temporarily the PAs were at one point going to be the solution to the staffing crisis. We no longer have a staffing crisis. – HA – DM 02 – Clinician manager**  The third thing, really I think is, there was also the context running up to that, we'd had a number of visa regulation changes, which had dramatically changed the overseas supply of doctors. When I first went up to [HD], I was the tutor in medicine, and so did the recruitment for trainees, and they were recruited locally, the SHOs. And the first, we had some like 36 posts, I think, in the first round I did. It's 600 applicants. And probably 450 were from overseas. The regulations changed and we were lucky to have sufficient applicants for the number of posts. – HD CM 02 – Clinician manager  I think the job market fluctuates, doesn't it?…I had the time where only people who were trained in Britain would work in Britain… and then we had the European Union..…and then of course in the run up to Brexit that died down, and then the people who come from elsewhere, there would be Visa restrictions.… So, I suppose it's a bit like investments, isn't it? I need a big enough portfolio that if one bit tanks, I've still got enough staff, that it'll all be fine. So, I've got a big enough team now that if no SHOs turned up because they're all on strike, I would cope. And if no PAs turned up because the government decided that the PAs couldn't work, I would cope. I've got enough doctors or nurses, so I kind of made book. – HE – TA 04 – Clinician manager | - Recruitment trend shift is noted in all sites particularly in response to broader labour market fluctuations - Despite not all sites having direct links to local PA training programmes, there was a consistent observation of increased availability of PA supply | - Different sites and units had different recruitment challenges originally, for example HA in urban city faced challenges due to high cost of living, whereas HB, HC and HD struggled with relative unattractiveness compared to the nearby urban city - Some specialties were less attractive to doctors therefore needed to consider alternatives - Sites also varied in how they compared PAs to other roles in terms of staffing models, some compared directly with international medical graduates, some with FY2 and SHOs |
| **Macro – National or regional workforce policy** | - Some organisations began developing and recruiting PAs due to initial guidance, support and pump-priming funding - There is currently widespread **confusion** over regulation, governance, and role clarity, and organisations feel limited in their ability to develop PA roles without national standardisation - Lack of registration and prescribing rights was a recurring barrier - There is a desire for stronger national framework to support PA role development - In some organisations, advanced practice roles have gained better support because of guidance from NHS England and elsewhere as well as their ability to prescribe | **Initially, the barrier or challenge was around their pay, and the job evaluation and what that job description should look like. Even the designing of this procedure that [Person]'s written, I do think there should be more national documentation to support this role. If we really are committed to it as a UK, then I think we should be more supportive of them. I think there's some national decisions need to be made, and they need to happen. It's not something that the Trust can do or change. – HA – CM 01 Manager**  ‘The question I had then, and it still remains the same question now, it's the same comment I do now, they are not a mandatory registered health care practitioner group. Therefore, they can't prescribe.… We couldn't use them that way because they can't prescribe, therefore they can't do some things...And they have been pushing for it for 10 years actually when I was talking to them. But they're still not registered and they're not registered now, so they still can't prescribe drugs’ - HA – DM 03 Nursing manager  I think there may have been a stream of funding from NHS England or the clinical commissioning group to promote this particular workforce. And it was the deputy medical director, it wasn't the divisional medical director. And he wanted to introduce this particular role into the medical workforce. – HC – CM 03 Manager  Because I think there's a lot of questions around scope of practice, et cetera, at the moment, which is up for debate. And I think that's limiting employers in their willingness to employ a PA, because if the scope of practice is scaled so far back, why would you employ a PA over an ANP, for example? It doesn't really make any sense. – HD TA 04 - PA  I don't think we can really do anything until the Leng review comes out. I think everybody's waiting for that. So I wanted to get some core governance in place, but in terms of making decisions around further deployments or employment scopes of practice, anything like that, I think we need to just wait for the Leng review. And I hope it's helpful, I hope it gives some clarity, and I hope it's clear for individuals, organisations, but also the wider bodies that are charged with standards, et cetera, around our professions. HE – CM 01 – Clinician manager | - Regulation and prescribing rights are shared concern between different organisations | - Due to the timing of interviews (prior to Dec 2024), participants from HA experienced greater confusion around regulation - In some organisations such as HB and HC, advanced practice roles were more established and seemed to mention more strategic engagement with national and regional policymakers |
| **Macro – Views of external stakeholders and collaborators** | - External stakeholders have played a mixed role in shaping PA role development - In some case early support from charities and private sources helped some organisations piloted PA roles - The lack of engagement from selected bodies like medical royal colleges - Currently awareness of rapid development from key stakeholders such as the BMA, RCP and public media/social media debates have partially impeded new PA development in some places but not universally | I mean, the colleges aren't... At one point I approached the Royal College and said, "What are we going to do? You've got eight around the country, they've got no curriculum, they've got no portfolio, they've got no access to anything, and yet they're supporting services in OBs & Gynae. So the college at that point wasn't even aware that they had PAs in OBs & Gynae. – HC – OT 13 Clinician  **So in terms of that noise and everything going on around it, our only concern would be, what impact is this having on the 21 PAs that we employ, and what support are they getting around that? … I don't think (the noise) would impact our decision to recruit more or less, or change how we do it, it would just be a case of, okay, we've got a vacancy, what is the best role to fill that vacancy? – HC – CM 02 Manager**  **Because of the media, right and also, both locally as well as perception nationally, it's been really difficult for PAs… our recruitment has been low, low in number because I think services have been worried about whether to employ PAs because of all the negative coverage. – HD CM 01 – Clinician -manager** | - All sites acknowledged external influences whether through funding, guidance or public debates - Awareness and concerns about BMA and RCP positions | - Types of external influence differs, some benefited from financial support and others struggled with lack of guidance and recognition - Impact of BMA statements was uneven where some organisations were unaffected (HC) and others were more cautious |
| **Meso – Characteristics of hospitals and departments** | - Organisations benefit from linking with PA training programmes to develop and employ, and training programmes at times created opportunities for role development - Some specialties struggle to attract doctors and nurses, prompting considerations of alternative roles like PAs - All the organisations currently face financial constraints that hinder any role recruitment including PAs | [Specialty A] is a speciality we find it difficult to recruit into the training grade or the junior clinical fellow grade or senior clinical fellow grade so that’s one. We didn't have the luxury of having a loyal workforce, in a manner of speaking, that would stay committed to the establishment or the organisation and it was quite expensive for the amount of support the junior clinician fellow required to actually train them up to a good standard and then they would leave the organisation and then we'll start all over again. – HC – DM 02 Clinician  [HD] were really trying to push the profession into various specialties and make good use of the students coming through and retain people in the area. So they were working quite hard to create job opportunities and if there was some sort of specialty that you particularly enjoyed as a student and were interested in, if there wasn't already a job opportunity there, they'd go to that department and try and see if it was appropriate, if they had use, if it was going to be worth putting time and money and effort into creating a job in that department. – HD TA 06 - PA  I think the reason it's worked is because [TRUST E], is we're co-located with the medical school, and the PA course was obviously one of the courses in the medical school. So we've been providing PA education as part of our education contract for quite a long time. So I think as clinicians here became familiar with the PA role because of seeing students come through, that then opens up the possibility of employment. – HE – CM 01 Clinician manager | - Recruitment challenges are common across organisations and units - Financial pressure seems common across organisations too - All organisations have some linkage to PA training programme though not all unit host PA students | - Organisations with stronger links to PA programmes feel more urgency to recruit PAs but struggle to do so for example HB and HE - Some specialties were less attractive to doctors therefore needed to consider alternatives - Hosting of PA students varies by units which affected role development opportunities |
| **Meso – Local experience and evidence** | - Previous experience of PA employment in other organisations influenced local role development - Positive experience with PA as well as student placement encouraged full employment in some teams, whereas negative experience discouraged so - Teams were often more receptive to employing more PAs when satisfied with the existing PAs | ‘And we recruited, I think, about 28 PAs at that point, we placed them in different trusts in England, and I sort of lost touch over the years. But certainly for a long time I was of the understanding that they’d all done really well and that the trusts that had taken them in had expanded the numbers that they made available for PAs, and they were looking to grow that role.’ – HA – CM 03 Manager  Some of it is seeing the value. Once you’ve got one and they’re really good, you go, “Great, let’s get some more of those.” – HB - CM 07 Manager  **And I think this is why I think the [specialty X] department had one issue with one PA and since then they're never recruited. So this is what it stems down from, and they really struggled. So I think that's why the recruitment from the [specialty X] side, they've never seen a PA ever again in [specialty X]...But the positive is like ..I think the ones that work well within certain departments, that they've experienced first-hand what we can do, they are more likely to hire – HC – OT 02-08 TB 01 – PA focus group**  And with the [specialty] department as well, it was, they had this entire service that was growing exponentially because of the amount of patients that they were being referred to like [specific] Pathways. So they needed an entire new workforce as well to make that pathway happen.…they had a physician associate permanent that used to rotate between the ward and clinics… it got so busy that they then allocated their PA just to do that…So they then hired more physician associates because they saw the benefit of having them on the ward and in the clinic and there was lots of reasons. – HD TA 06 - PA  ‘I think that’s right. So, I had one who proved to be outstanding, and so I decided if I had more like her that would be very worthwhile. So, I had seven quite early on, and I remember at one stage I had more PAs in the ward than most hospitals had PAs..’ – HE – TA 04 Clinician | - Positive experiences with PAs encouraged further recruitment - Those who employed more PAs consistently described them as useful however none had documented evidence of effectiveness | - Variations in how much evidence was drawn on: some relied on international evidence, some on internal organisational information or regional evidence - Some units had negative experience with PA students and chose not to proceed with PA role |
| **Meso – Organisational leader or champions’ perception** | - Many organisational leaders initially had limited or mixed understanding of PA roles - Perception of PAs varied also vertically across organisational levels, with inconsistency between clinical and operational leadership. General managers viewed PAs through an operational lens, while clinical leaders focused on service safety - Champions, especially clinicians at the unit level, played a key role in developing PA roles - Some are supportive of PAs as a distinct profession, whereas others viewed them as another occupation group, emphasising instead the importance of recruiting the right individual and designing roles to their skills and providing training to those skills | **The clinical directors who were medical professionals in the organisation wanted (PAs) within the…that's what we decided would be best, and they were really up for supporting them in the organisation. The blocker that we got was the financial position, and that's where it stalled unfortunately. So we had support from the clinical all the way up to the executive medical director. He understood what we were trying to do and that's the reason that we tried to pursue it. – HB – CM 09 Manager**  I think there may have been a stream of funding from NHS England or the clinical commissioning group to promote this particular workforce. And it was the deputy medical director (who) wanted to introduce this particular role into the medical workforce. If I'm honest with you, I have my opinions, I expressed my opinions at the time. I was told I was being a nursing snob. – HC – CM 03 Nursing manager  It's very much the consultants who were the main champions for it. And then just how it works in terms of process, I was the general manager at the time, so I had to match a business case, take it to the executive and prove the financial benefit, but also the patient outcomes, the quality side and the workforce benefits. – HE - DM 01 Manager  If that's all we want, if we wanted just someone who can reduce the workload of consultants, then we needed the other type of PA, the personal assistant, who I can train to my requirement, and who does those sort of things. Now, in reality, that is actually what we did. We trained these individuals who come with a certain level of qualification, to what we need. We maintain that training and skills, and we maintain that oversight and the check in and to the quality assurance, the governance, over that whole system. That's how we saw the utility within the department, and we have invested but also fostered that relationship, so that they find it easy to come to us if they get to know their limitations like any clinician does. We all have our strengths, and so this is a relationship that's been built across many years. – HD DM 01 – Clinician manager  There is an awful lot of medicine that needs to be done, and so the more properly trained hands-on deck the better. And I'm not really that bothered about the roles. So, I'm not so wedded to the PA as a great conceptual advance. But if you choose the right people, they're excellent and they'll practise safely and they'll extend what you can do. And so if there was funding and there were good enough people and there were roles to do, then I'd be more than happy to pick them up. – HE – TA 04 Clinician | - Champions were crucial for PA role development across organisations - Mixed or unclear understanding of the PA role was widespread across organisations too - Units with employed PAs generally had more supportive leaders | - Perceptions differ by roles, as general managers viewed PAs through an operational lens, while clinical leaders focused on service safety - Some organisations had consistent support across leaderships, others experienced internal conflict or resistance such as from nursing leadership |
| **Meso – Organisational need and planning** | - Service pressure such as staffing shortages especially at lower tiers, or need for service redesign due to external reviews prompted consideration of PA roles - Organisations are also moving towards diversified, multi-professional teams - In some organisations, ACP roles were prioritised over PA in planning - While most organisations and units did not have routine workforce planning, PA roles were sometimes introduced in a 'wait and learn' approach, especially at the unit level - PA role decisions were often made at unit level with limited awareness at organisational and individual level - Financial considerations often outweighed strategic workforce needs | I wrote the business case for the first in trust PAs a number of years ago, supported at that time by the director of medical education, because our training was in crisis, and we were also spending a lot of money on locum, short term locum, FY2s for the ward. And so we put back together in a cost avoidance business case, to appoint the first in trust PAs, to improve the quality of our training, improve the quality of care provision on the wards, and to reduce costs. – HA – TB 02 Clinician manager  So the attraction to me, for PAs, is that they are trained in the medical model of diagnosis and management, and I felt that would…because I wanted them to be on the wards mainly. And I made that very clear to any PA who came for the job, because we interviewed PAs over the years who spend most of their time in theatres like an SCP. I'm very clear to them that is not this role. We have SCPs, that that's a separate role for us. This is about being on the ward as part of a team, working in the pre-admission clinic as part of a team. And so that is why, because I wanted them to work with that team, I want them to be trained in the medical model of diagnosis and management. It's not to say I don't think ANPs can do that role, they can, I worked with them at [name]’s when I was training. They did it brilliantly. I just felt it would fit best for us. – HA – TB 02 Clinician manager  if we'd had our choice, I'm sure it’s probably still true today. If we had the funding available, we'd go for an ANP just because really rewarding our nursing staff, that'd be the main emphasis, not necessarily that we'd think on a day-to-day basis one would be better than the other in terms of their clinical delivery, but more in terms of the long-term relationship, and commitment to the service. – HB – DM 01 Clinician manager  I don't think anybody has a handle in workforce planning. I don't think they really have been part of any sensible big plan. Certainly any workforce planning that's been done locally with us has been at a departmental level, it's because we had them as interns and we thought, oh, this might work quite well. And we can't get any more junior doctors because there's no more funding from NHS education. But we could apply for some rolling funding for PAs to help support that workforce, and that's really the level of planning that got them in – HD TB 03 - Clinician  **Acute medicine did three years ago now, and they wrote a business case, and that business case was largely around flow. So it was largely around by relieving the junior doctors, you can improve the flow, so you can really improve your clerking of the acute medical take, the task based work that it takes...So that was the essence of the business case. I think, like I said, we kept it going afterwards. We showed that those benefits were realised. – HE – CM 01 Clinician manager**  I got my PAs earlier during PAs when it was difficult to recruit doctors and I looked after them carefully. And so, I've got lots of dividends now being repaid by looking after them, and that we can use them for various niche things.… So, my way of thinking of them is think of it as a portfolio, like a financial portfolio. And so, they're all my stocks and shares. I've invested in different areas so that if one thing goes wrong, I'll cope. It's a bit like on the doctor front, so I've got training doctors, and I've got doctors from abroad and who aren't part of the BMA. The BMA go on strike, half of my doctors won't go on strike, and none of my PAs can go on strike, so I'll survive. Whereas if I had all of one sort and they all went on strike, I would be in big trouble. – HE – TA 04 Clinician manager | - PAs being envisioned as supportive, administrative roles that improve continuity were common across organisations | - Organisations varied in whether workforce planning were being officially conducted, with some had structured business case processes, and others more trial-and-error approach (driven by financial factors) - Prioritisation of PA vs. ACPs differed between organisations and units - Also variation in terms of what the ideal ratio between PAs and doctors are (e.g. HA TA mentioned 8-10 doctors to one PA, whereas HE TB said 1-2 doctors to one PA) and thus what PA actually do |
| **Meso – Organisational policy and arrangement** | - Business cases were commonly required to justified PA rol§es in some organisations, however with financial viability being the main focus - Governance structures for PAs were often created retrospectively and are now under review in many organisations, and not well known across team leads - At the unit level, clinical team members were often unclear about workforce decisions and these governance or workforce documents, suggesting that communications around these roles are inconsistent or lacking | It has to go through a business case. So like in any trust, you will have to go through a process…we put forward a business case also within division, working out that we'd ideally like a physician associate on each ward. Now, we didn't get fully funded for that, just because it was coming off the back of COVID. – HC – DM 03 Clinician manager  I think that it was very, the idea was quite open, you know, you can get them to do what you need to do in service, you know, in your service. But we, at that point we didn't, we didn't discuss you know, clinical governance and all of that because, and I would absolutely say that clinical governance had only really got off the ground in clinical environments you know, a few years before. – HD CM 01 – Clinician manager  One of the things we did as an organisation a while ago was we really went over a PA governance policy. And I worked with a senior PA to do it. We should have had one long before, to be actually honest with you. It was an organisational oversight. But we wrote a very comprehensive document that set out the PA role and went from everything from recruitment, right through to revalidation when it comes. That spelled out, so when we are recruiting PAs as an organisation, this is our position, this is what we look for. When we induct a PA in the organisation, this is what we look for, then let's go through all the elements of the role and say exactly what can and can't be done, and the governance around it. - HE - CM 01 Clinician manager | - Financial justification was a central factor in PA role development process across most organisations - Governance documents for PA roles were often under-development in organisations - There was not enough evidence to suggest clarity and communications among frontline staff was achieved when introducing PA roles | - Business case requirement and approval processes varied, whether it was required or not, and whether it was reviewed at divisional or organisational level (partially due to some organisations are under financial measures) - Governance structure and policy also slightly varied in terms of whether PAs were previously under medical or nursing management and whether PAs were under advanced practice guidelines - Some clinical teams, for example HE TA, may have communicated effectively with the clinical team members |
| **Meso - Resources and processes for role development and recruitment** | - Funding sources for PA roles were unclear in most organisations with many unable to identify exactly where the money came from, including unit leaders, as managers often inherited PA budgets without clarify on how they have been created - External funding such as from ICBs, NHSE played a key role in stimulating PA role development, however this could have led to external driven or superficial motivations where roles were simply created to access funding - Financial difficulty and lack of funding is now a major barrier to new PA employment - Recruitment processes varied across organisations, partially linked to whether there were connections with PA student placements | ‘Now, there was talk of a budget for employing more PAs. The problem was there's been a financial crisis in the trust, and so that budget's sort of disappeared. I don't know what happened, but then as soon as we actually started looking for them, then there was a chat of like, "No, actually that's not happening anymore." So we were going to employ another six PAs.- HA – TA 02 Clinician manager  I think when we had, because we were really careful, I tried to be really careful who we appointed. And we didn't appoint the first time. I know we advertised two or three times, because we wanted…it's always better, isn't it, to wait to appoint the right person? Even though setting up an interview, it takes time and consumes time, it's much better to wait. So I think, we definitely advertised twice or three times, yeah. And the first two…and so I think, because I was aware that this might be a problem, I really tried to appoint the right people who I thought could address that. – HA – TB 02 Clinician manager  Obviously, all departments wouldn't have said no because I think there was a fund available and there was resource available so, okay, I think we'll have to take extra staff on. – HC – TA 05 Clinician manager  So I've got an establishment in my budget for roughly twelve full-time equivalent physician associates...there's always a bit of flexibility in your budget.… The establishment funding and [team A] for the PAs has been there since probably for the last ten years, and I don't know...obviously, I wasn't around when it was first introduced or when it was first decided that we reduce the budget in this way. I just came in and was told, you manage this team now and there's your budget for them essentially. So make sure you don't overspend it. That's what you're kind of told. So I've had quite a lot of freedom with that budget to play around with the posts. – HD DM 02 - Manager | - Financial considerations were consistently a pre-requisite for PA role development in all organisations - Current funding uncertainty was a common theme with many unsure of whether there could be new employment | - Funding sources varied with some organisations relied on external funding, and others used cost-neutral business cases - Financial status influenced some organisations’ capacity to further develop and recruit new PAs - Recruitment processes differed in organisations, some units conducted detailed multi-round interviews, and others (e.g. HB) had centralised organisational-level interview process at one point |
| **Meso – Views of other departments** | - Views from other departments did not affect whether PA roles were developed within specific organisations or units, which may be less relevant in the UK NHS setting - On some occasions, rotating clinical team members from other departments were more hesitant to work with PAs, but this did not influence recruitment decisions | But certainly with the numbers that we have, to be honest, all our fellows are very happy to work with the PAs. I don't think there is a lot of bad feeling, and they're happy to organise tests, do the tests. It's visiting junior doctors who are coming in who are trainees and just doing a couple of days with us who don't know the PAs, who are a bit more hesitant, but of course the PAs probably don't even ask them because they normally just ask one of the doctors they know. - HA - TA 02 Clinician manager | - N/A | - N/A |
| **Micro - APP individual interest and intention** | - Some PAs especially early graduates choose jobs intentionally based on positive experiences during student placement or at specific organisations internship placements, desire for shift / no shift work, having friends or colleagues working in the same location, interests in specific field, etc. - However more recent graduates mentioned they generally apply for whatever jobs are available | I know everybody applies for any job which comes, and I can't see that this is a viable option. Unless they have already predetermined what they want to do, how they want to do, and just follow that path. But when the jobs are very scant, then there's a tendency to join anything what is available. – HB – TB 04 Clinician  The way it worked when we first did it is that we had an intern year, which later was voluntary, but made sense when I always advised people to do it…It was very new, it was still finding its feet, so I did six months in [team A] and six months in [team B]. I really enjoyed my time in [team B], but found it to be much more of an admin kind of role…But I loved [team A], like loved it, so applied, got a job, and I've been in the [team A] then since essentially 2013. – HD 02  **There was always a handful of jobs going. I'd say at any point there was between two and four jobs. I was applying for everything hospital-wise. There were more jobs, to be fair, when it came to GP. And at one point after I didn't get any of the hospital jobs, I applied for a GP job. I got it, but it was through that process of talking to them and I was like, "Actually, I don't want to do this."... I knew I wanted to do secondary care, whatever it was. – HE – OT 01 PA** | - Across locations there is a shared experience and understanding of job market changes - In the past jobs were easier to find and there was flexibility to choose preferred places; whereas now jobs are much harder to secure | - Preferring jobs in organisations closely connected to PA training programmes were mentioned in selected organisations like HB, HC, HD and HE - Internship placements were only available in a small number of organisations, and where they did exist, they were closely linked to PAs’ own interests |
| **Micro - Clinical team members’ perception and understanding** | - Opinions or perceptions of the wider team were much more polarised, however they were not central to development and recruitment decisions, which were largely led by the consultant body or organisational and unit leaders or champions - Junior medical staff views were not necessarily considered, as they rotate frequently and are more concerned with their own training - At the unit level even with the consultant bodies, many were unclear about PA workforce decisions as they were made before their time | PA role was introduced on the [Team B] ward... It's been a good few years now. I can't think how old. Five, six years ago, something like that. It's definitely been a while. I guess we were all a bit confused when the role was brought in, like what it was going to add to the service, what limitations there were from having a PA and were the PA is going to reduce the amount of doctors that we were going to have. – HA – TB 03 Nurse  I had a spell of doing some PA interviews for new PA roles, not really understanding what they were. But then when we'd appointed those people, it became quite clear kind of, you know, what the role was becoming and I understood it as an evolving role, kind of nobody quite understood what it was and how it was going to fit in the services. But so when in surgery, I understood it as a kind of more of evolving role, trying different things and seeing kind of what works. – HB – OT 02 Other  So I remember them when they first started, because I think I had actually just started back in [team A] myself at that point. And so I kind of grew up with them, so to speak. And I remember there's a lot of anxiety about what they were going to do, so to speak, and how they were going to fit in. But to be honest, at that stage in your training, you just worry about your own training realistically, not really about anything else. – HD DM 03 – Clinician manager  ‘No, I don't actually. No, there was no resistance... I think everybody understood their role, just because of the departmental meetings that we have and we're very good at explaining what we're doing ...So I think everybody was aware of what they would be doing. - HE – DM 01 Manager | - Lack of understanding about why PAs were introduced was felt across junior medical staff and nursing team members | - Organisational and unit leaders who were directly involved in developing the PA roles felt there was a shared understanding (e.g. HE) but this was not experienced by other team members |
| **Micro – patient perception or preference** | - In most cases there was little to no evidence that patients’ views or opinions were directly considered in the development of PA roles - Occasionally, potential improvements to patient experiences were cited as one reason in developing PA roles | One of the reasons we developed [specific procedure] follow up programme, was because I felt patients weren't happy with the follow up we were providing.…they weren't happy with the junior resident doctor they saw last time…my secretary would say, “Mrs X rang. She didn't understand really what the doctor was saying.”…So when I developed [the programme], and it's run by a mixture of our ANPs and our PAs, sometimes I have a gap. Maybe one of my patients doesn't cut, and I'll say, “Shall I see some of the follow up?” And they sometimes said to me, “No, they don't want to see you.… they want to see the PA,” because they're in follow up for five years, a lot of them, and they develop a relationship with these people. – HA – TB 02 Clinician manager  I don't think (patients) necessarily have an opinion…I think they will have been told that, who they are being seen by you know, I'm one of the doctors, I'm one of the physician associates, but I don't necessarily know if they would register it or you know, or the general population if they would register it or…Maybe a very, very small sense you know, maybe a couple of patients said, oh I don't want to be seen by a PA. – HD TA 01 Clinician manager  **I don't think patients really matters that much….All people want to know is that they're getting the best possible care, that the person in front of them can tell them what's happening, why it's happening, and not to have to defer to somebody else…It matters more to us as professionals because we love hierarchy and lines and whatever, but I don't think…members of the public really just want to get the best care. – HE – CM 02 Nursing manager** | - Participants commonly felt that patients would not understand what PAs are | - In two units within HA, improving patient experience was noted as a minor contributing factor in developing PA roles |
| **Integration** | | | | |
| **Macro - National or regional scope of work and service reimbursement policy** | - National policy restrictions on prescribing were consistently mentioned as a barrier to PAs’ daily activities, adding additional burden to the clinical team - In absence of clear national guidance, with only restrictive directions from certain organisations, there was a lack of shared understanding about the scope of practice, leading to confusions and different expectations between team members, especially in teams where PA previously have been allowed advanced tasks - The lack of regulation in the past was a concern, particularly regarding accountability | **I think the big step forward would be the ability to prescribe medications and request radiology investigations. That would be a big output for everyone because it takes off the burden from the rest of the team, from the medical team…I see many times for example, we do the ward round together and they cannot prescribe the medications. They have to write it down and then ask another doctor to go back and prescribe. So this is all time consuming. – HA – TB 06 Resident doctor**  I don’t know how the, you know, where the Leng review is going to turn out, how the various political campaigns are going to turn out, but I think it’s got to a very unnecessary and unpleasant stage.… I think I’m just keen that the GMC move ahead with that, getting fully regulated.… I think it’s really just more from a governance framework point of view that we know we’ve got that independent oversight of practice...I think we need a bit more of a national steer and national guidance about how to do that safely without causing too much distress for the individuals – HB – CM 06 Clinician manager  I think there was no national consensus what we can or what we can’t do right at the beginning in terms of advanced procedures. I think that’s where the different opinions varied, and I think that’s where there’s some peers that do things and some peers that actually don’t do things in different hospitals. – HC – OT 02-08 TB 01 – PA focus group  Where it's become complicated, is as different groups and colleges and the BMA have started publishing scope of practice guidance. We've had long conversations with the legal people. Is, what's that mean? And whilst those things have no authority, they become something that…Now 15 colleges have all released different guidance, which are all conflicting, and the BMA have released their own guidance, which is conflicting – HD CM 02 – Clinician manager | - Lack of prescribing rights is consistently raised as an issue - The absence of official regulation was also a concern especially among junior clinical team members | - The challenge of managing undifferentiated patients was highlighted by some team members in selected units |
| **Macro – APP representation outside of organisations** | - Overall negative media bias was noted on social media and general media platforms. PAs were often descried as being “attacked”, and such coverage were described as tiring, exhausting and demoralising - Although many individuals did not actively follow social media, there was a general awareness of these negative accounts - Some of the anger expressed by resident doctors is not necessarily directed at PAs as a profession, but reflects broader dissatisfaction with medical training - PAs reported feeling they had to be extra cautious when working with colleagues because of these, some PAs chose not to promote themselves externally due to fear of negative reactions - External reporting of PAs influenced some organisations to clarify or strengthen internal governance policies - In some organisations, PAs were asked to pause working on certain procedure or weekend work due to concern about external scrutiny | **‘We do read those bits, and it does affect how when you are working, then you have become conscious of how…even if you are not treading on negative lines, you relate to everyone the same way, but when you read that, it does affect your psyche a lot a bit. And then when you come to work, you are cautious not to tread. When you see anonymous opinions on…although as professional, it should affect you, but we are humans, it does affect how you approach things. It's no longer, I think, initially felt you have to be cautious of who you ask to prescribe for you or request imaging - HA – TA 01 PA**  So we sort of got taught how to do [specific procedure], all of these more advanced procedures. And obviously, because I've been here for much longer, I've been keeping a log of how many I've done and I've got to a point where I was sort of able to do these. But then with the BMA and all the sort of everything going to court and people being sued…just to sort of avoid any sort of backlash, we've sort of temporarily paused that, which has meant things get delayed on the ward. – HC OT 11 PA  …I've very much tried to tell the more junior members of my team to not go on the internet and look for our job role because it's just not worth it. The stuff that you read is horrendous, and it makes it sound like everybody hates us. And it's trying to block that bit out of your brain with people you work with that think that we are worth our weight in gold, and it's to separate those two areas of your brain. because it's so easy to fall into a dark hole and you just keep going and going and going…I've been involved in going to our line manager to be like, "We need to support this person. This person needs more than what I can offer as a friendly shoulder." – HD TA 02 - PA  I know everything going on in the news at the moment won't be helping…The physician associates in our department, they do the induction for the junior doctors.…with everything that's been going on recently, they've been a bit hesitant to even do the induction in certain cases. – HD TB 02 - PA | - Nearly all PAs chose to withdraw from social media and found online debates tiring and exhausting - Most organisational leaders also reported experiencing negative encounters including responding to critical media coverage and FOI requests - There was a common perception that immediate team members were generally supportive | - In some organisations, PAs’ clinical activities were reduced, particularly around advanced procedures and weekend work (HB and HC), or PA reduce their full time work (HD), while in other sites no such changes occurred - Senior leaders’ responses varied, as in organisations with larger numbers of PAs, leaders were more proactive in offering support and advocacy |
| **Meso – organisational culture** | - PAs integrate more effectively into organisations and units that already have PAs in place, are familiar with the role, and operate with less rigid hierarchies - Positive cultures were described as flat and collaborative, less hierarchy, good relationship between doctors and nurses - Recent negative media coverage has made the organisational climate and team relationship more challenging in some settings | I think the key thing is that the [ward], we’ve tried to make it a very flat hierarchy because we want to sort of be able to work well together. And I think there’s quite a good atmosphere and that’s why… It was my predecessor who was very good at all this, so I can’t take the credit, but there is quite a good atmosphere…I think the key thing is the nurses and the PAs and the doctors all get on relatively well…There are nights out where they all go on nights out together and stuff like that – HA – TA 02 Clinician manager  I think we are a really small department. We've got…so I find that if I want to speak to someone about them, I can find them, chat to them, explain what we're doing. Obviously in a larger department that might not work. But so I didn't feel that sending out an email announcing them might be the best way forward. And I think I don't want to underplay concerns there. I'm sure, I think there were concerns about the role, about whether the role would encroach onto the SCPs, onto the AMPs. There were concerns about the funding level. I don't want to underplay that, I think there were. I think I might have tried to have conversations about why we were bringing them in, and I think the PAs themselves helped me a lot by being who they are, as well. – HA – TB 02 Clinician manager  I’ve asked two physician associates in our department to lead on the morbidity and mortality because I know they’re a constant in our department. I know that they’ll deliver it well. As part of that though they’re not able to, they don’t feel confident to ask a junior doctor to say we need you to present our mortality and morbidity meetings. So they’ve lost that level of confidence. They don’t feel like they can approach a medical…- HC – TA 09 Clinician  I think as a department we're very good at, there is no hierarchy and everyone gets called by their first name, and everyone gets made fun of or joking about with consultants who are in their 50s, talking to people who had just started the job at 17. I think we're very good at doing things as a social aspect and acting like basically a family, and anyone who voices any concerns is taken very seriously, whether it's healthcare support worker or a medical staff. So I think it's a different bridge between nursing and medical staff here, because I think there's very much less of one, but the PAs then tend to be in both groups because we're dragged away when maybe they don't want to bring it up to a registrar about what's happening, but they want the help with something, which I'm happy to do. – HD TA 08 - PA  **So there’s definitely something about, if the clinical team embraces the PA role, really brings the PA into their team, very clearly defines their scope and their responsibility. We definitely have PAs who do better in teams like that, and they tend to be my good teams. So they tend to be the teams that I have more confidence in anyway, that have good outcomes, that have an engaged consulting body who work together well. – HE – CM 01 Clinician manager** | - PAs were generally better integrated in more collaborative and flat organisations | - In some units recent negative media coverage has made the organisational climate more challenging such as in HB and HC |
| **Meso - Organisational strategy and planning** | - In some organisations especially at the unit-level, there was clear planning for integration linked to the rationale for developing PA roles (sometimes tied to the business case) - In other places there was no shared understanding of PAs’ specific roles, instead the role is supposed to evolve through trial and error - Organisational / trust-level planning appeared weak, partially because PA numbers were relatively small and little forward planning was dedicated to this specific group | Because although we appointed the first in trust, they've since gone into other departments. So there would perhaps be a wider knowledge that these are starting in the trust, so we did that as well. And I tried to do a lot of groundwork, not necessarily informal meetings, and obviously it went through our CSU, but just having conversations saying these people will be starting, but won't be doing this, or they will be doing this and this is why we're employing them. – HA – TB 02 Clinician manager  **I don't think the medical team had figured out what the PA was going to do or the PA, you know, I'm not sure that they'd figured out that role… I think probably where it's worked, there's been an understanding of what the role is, there's been a gap to fill, and so everyone is motivated to make it work. But if there is a bit of a not really sure, then perhaps, you know, with [unit X]…they're quite complex environments to be in.. – HB – DM 02 Clinician manager**  We also knew that PAs tended to work in one area and stay in that area for a long period of time. And so we immediately saw that there were some areas where that could become really, really valuable, because, to give a example would be, we had a PA that was interested in [condition] and the [condition] clinic. Now, you've got a really focused area of clinical practice where your doctors in training moved through a rotational basis, and there was a real opportunity that there's a small set of extended skills that you can gain and an experience over a long period of time and provide stability of expertise in small areas. – HD CM 02 – Clinician-manager  We consulted really, really widely on it. It took a long time, because we were getting lots and lots of stakeholder feedback. We took it through the medicines optimisation group. We took it through each divisional governance group. We really did a lot with it. But at the end of it, what we asked departments to do was to make sure that first of all, they knew every single PA, they knew exactly who their supervisor was, and they kept a register of that, and they checked on having their appraisals. And they had a really clearly defined scope of practice for each PA. – HE – CM 01 Clinician manager  I can tell you roughly how many nurses we have, how many therapists we have, how many midwives we have’ I can't tell you with a hundred percent surety how many PAs that we have, because i’ doesn't really make it to any numbers ’hat we're looking at…So we know how many consultants, how many junior resident doctors, whatever, but as a group’ and we're only talking about it last week, we've got this really skilled group of people who we don't really talk about, know about, count. – HE – CM 02 Nursing manager | - The planned or evolved role of PAs was tailored to unit needs, with variations between units within the same organisation - Across sites, PAs’ roles often included some administrative components | - In some organisations and units, there were little to no strategic planning prior to employing PAs (such as HB) and this continued to create difficulties - Other units reported more deliberate planning process which could have supported better integration and role development |
| **Meso - Organisational policy and arrangement** | - In many organisations, policy and governance documents for PA roles were created retrospectively and are now under review in several places, which some mentioning the process took a long time - Many organisational leaders, managers and clinical team leaders were not aware of fully absorbed these documents, therefore these policies did not make a noticeable difference in terms of integration, although this issue was not unique to PA roles - In some organisations, centralised governance document did not fully capture advance tasks PAs are taking on - Induction process for new team members in terms of introducing PAs varied considerably between units-specific arrangements such as workplace location (e.g., office), rota management (often influenced by PA numbers) and uniform were also highlighted | ‘Saying this is the formal structure for our PAs and what we expect from and everything. It was a lot of words and it was probably basically fact, but what wasn't there from my perspective, it wasn't clear who was going to be doing the reviews and the assessments and who was reporting to who and so on. So it was fine as far as it went, but as far as I'm aware and I do miss stuff because I'm not on all of the mailing list because I'm not seen as an [specialty A] consultant, as far as I'm aware, I don't think we've got anything more stretched in place, but they are now having to use the [Hospital A] appraisal system.… We're very bad as professionals, probably that's the right word, at actually dealing with poorly performing colleagues, just in general, because it's always difficult to call them out, and I wouldn't say that that's unique to PAs, I have to say.’ - HA – TA 07 Clinician  because we were the first and, obviously, we had to set our footprint and tell people who we are and make a lot of protocols. And so because none of the protocols were based for us, we had to create all of these things ourselves. There wasn’t even a set uniform for us. They hadn't even decided what uniform to put us into. This is what I'm saying. We had to lay the groundwork literally from ground zero and now we're in a place where we're a little bit more recognised. This is a lot of work, don't get me wrong, but we lay all the initial groundwork... There was no legislation as in proper documented things. We had to sort of work with different teams to sort these things out and make changes and additions and put new protocols in place.. - HC – OT 10  What I've struggled to understand at times is whereas we do have the core scope of practice, and our colleagues have developed in some areas quite an advanced skillset and scope of practice, what I struggle to understand a little bit about right now where I'm at is how this has evolved in the way it has, and we are left in a situation where some of our colleagues feel very exposed that they are practicing in a certain way, but perhaps there hasn't been that tighter governance around this role. And I think that's quite unfortunate…what I'm sometimes struggling to understand is how we got to this point where we are now almost going back and saying, "How did it happen that you're doing these things when actually there hasn't been the structure around that?" And I think what would be part of my work moving forward is, is how we, how we make that safe for them. How we make them feel that they are okay to do this work, whether that is based on what the organisation decides as their employer, how that fits in with the guidance nationally from the Royal Colleges…from the GMC. – HD CM 03 – Clinician manager  We wanted them to be part of the team, so we put them in exactly the same scrubs as our consultants and junior doctors. We didn't want them to feel different, I guess, so it was very much how do we make them look a part of our team? - HE – DM 01 Manager  **I didn't feel there has been significant change since (the governance policy), apart from there is now just a document that supports their role. But everything that they were doing, they are still doing. There's just a documented policy to back it up. But actually, like I said, the engagement, particularly within our three surgical specialties have been so strong towards PAs from the very beginning, that whether that policy was there or not wouldn't really made much of a difference to how they were integrated within those teams. I guess what it probably has done is created some awareness for the rest of the workforce within the organisation. So maybe that's in colleagues and provided them a bit more understanding of what that role is and how they can work together with them. – HE – DM 01 Manager**  I know the people that I know contributed to (the governance framework), did that, worked really hard alongside with the chief exec.… it was nice to have that framework definitely, and something that we could go back to. Did it change my practice? No, because I think we were working towards that framework anyway….But it was just nice to have it in a formalised framework that we were all like, this is why we're doing this, and this is why we're doing that…I'm sure (the governance framework) was sent to lots of people and I'm sure it was spread by far and wide. But did they, everyone open their inbox and have a look at them and read it? I don’t know...lots of stuff is sent to people at NHS inbox constantly, and it's just, everyone's busy. – HE -OT 02 PA | - Governance documents for PA roles were often still under development across most organisations | - The level of formality and clarity of policy and governance varied, often depending on the size of the PA workforce, in HD this is more developed - Induction process for new team members in terms of what PAs do varied, where some had clear and deliberate effort of clarifying, and others considered PAs already part of the norm (e.g., similar with pharmacist where no separate introduction needed) - While PAs typically worked in doctors’ offices, differences were noted across units regarding uniforms (how different with junior doctors) |
| **Meso - Resources to support role activities and integration** | - At organisational and unit level, time and resources are required to develop governance structures as well as role activities for PAs, but this has not been as systematically addressed and fully developed as ACP roles - Supervision for PAs is often not written into clinicians’ job plans, creating difficulty in providing dedicated support for integration - Limited resource for physical spaces and cost associated with improvement such as uniforms / scrubs | This is all about time and money, isn’t it? Time, money, and motivation. If it’s a role that you’re truly motivated for and think is positive, you’re willing to put time into it that might not necessarily be recognised in your job plan or whatever. Now, as far as postgraduate training is concerned, I get funded to pay consultants to provide postgraduate support for postgraduate trainees in medicine, but not for ACPs, not for PAs. So, there’s a kind of general understanding that, you know, that postgraduate funding blurs and merges to do all of those functions. So, if you haven’t got someone who’s motivated to do it, then you’re kind of – so we kind of recognised that we had a very motivated consultant colleague who supported the training and supervision of the PA, and I think that’s really why it worked well. – HB – DM 02 Clinician manager  And I think I've seen how well our department supports the PAs, the fact that they have proper supervision, the fact that they get dedicated teaching and learning time, they get dedicated administration time as well. And I think I've seen how well we look after them as a team and how much they're valued by the senior clinicians. – HD DM 02 Manager  **When I first started, we had a reputation or I had a reputation for managing the PAs since about 2014, 2015, and so I used to get requests for like, “What is it like to look after them? How much work you do? How do you make them work?” And we had a few visits. I remember one who visited from somewhere that shall remain nameless. And well, they had had this great idea that A&E was a bit inefficient and they thought they would improve it by having two PAs run their A&E as their junior doctors, and it has been complete and utter unmitigated disaster. It was very difficult not to laugh at them for being that stupid. And so, I think people don't understand quite how much work you have to put in. – HE – TA 04 Clinician manager**  I think starting with the scrubs, we’ve said in this trust, you think it would be a good idea to have now name badge, it says my role, but we think it’d be a good idea to have a colour. But actually in this trust, like no doctors or levels wear different colours…So your name badge is the main thing, but is that enough? And we as PAs have been saying that, not just we’re saying that all the time. I know it’s been worked trying to organise that, but it’s difficult one. Where’s the funding coming from? – HE – OT 02 PA | - There was broad consensus that time and resources are needed to continuously support PAs’ integration, but also recognised as difficult to sustain | - The presence of a dedicated lead PA and associated resources to support role integration varied between organisations, only HC and HE had so - Physical space constraints were particularly noted in inpatient ward specialties - In some organisations such as HA, resource challenges were not as prominent, which may reflect the smaller number of PAs and also unit-level resources are more available |
| **Meso - Clinical training resource and opportunities** | - Some participants raised concerns that the presence of PAs limited training opportunities, from FY doctors to specialty trainees - In certain cases, the issue seems to appear less from the presence of PAs but more from junior doctors being uncertain about what learning opportunities and exposures they should be getting, and feeling they needed to ask for them - Senior leaders offered a contrasting view, suggesting that PAs can actually enhance training opportunities by taking on routine tasks, and at times directly supporting training for junior doctors - In some teams, conflicts over training opportunities were resolved on a case-by-case basis - The extent of this issue varied between wards, especially the specialty context | I can understand why people might be worried about PAs taking away their educational opportunities in other units, particularly when it comes to performing procedures. I think that's probably going to be the thing that junior doctors are shortchanged on. If for example, you've got PAs that do all the acidic taps for patients who need acidic taps in the hospital and they struggle to find opportunities to practice. That hasn't been an issue where we are and where we work because the PAs don't tend to perform procedures. So there are plenty of opportunities for people to practice. - HA – TA 03 Resident doctor  So I haven't attended any clinic or [specific procedure] or referrals on this rotation, but I haven't asked for it, to be honest. But I do know that (PAs) do get those opportunities. I wouldn't say that they're necessarily taking them away because I haven't asked for it. So I don't know how difficult it is to go. I do know that some of the other foundation doctors, the FY1s, they have gone for like different ward work, like so on the enhanced high care ward, which is bit of a different experience. I would say that (PAs) do get more opportunities than me, but I'm not sure if they're taking them away, because I haven't asked for them. – HB – TA 01 Resident doctor  I just don't understand that they're stealing the opportunities because somebody's obviously authorising that and there's plenty of work for both. On the one hand you want to develop your service so that you, so that the registrars can do that or whoever can do the better or bigger or more complex or whatever stuff that maybe doesn't come around so much and they're doing more of the humdrum stuff. But as far as, I just don't get it, you know, stealing training opportunities. But that's my own thing, I’m not saying that doesn't happen. I just think if there are trainees and they're not getting access to do the training that they need to do to become senior trainees and consultants and because of a PA that just seems bizarre to me, how that situation has arisen and it smacks to me of. – HD TA 05 - Clinician  **We have a very skilled PA in [specialty X] here who's been trained up to assistant in [specific procedure], which is one of our big income generators. Now, it means that the junior doctors can actually be learning how to do the [specific procedure] rather than standing, holding the kit and caboodle, which is the bit they don't like, but the PA is able to be trained to do that. So that's been an example of something really successful, but there's definitely been areas where it just hasn't really worked. – HE – CM 01 Clinician manager** | - Concerns about PAs limiting training opportunities were mentioned in all sites from medical trainee | - In some clinical teams like HA TA and TB, and HC TA, PAs did not participate in procedures or there are very large number of work / opportunities, thus concerns about training opportunities did not arise - In other settings (e.g. HC TB), where PAs were more senior and undertook advanced procedures, they were able to support junior doctor training |
| **Meso - Local experience and evidence** | - Many units with PAs reported positive experiences, including improved team continuity, stronger relationship and better patient communications - In some places, official audit or evaluation of PA roles had been conducted, but these were not consistently cited, disseminated or shared across teams, and feedback was limited - Participants highlighted the challenges of demonstrating PA impact in the current climate - Negative reports about PAs often stemmed from anecdotal cases or broader professional debates rather than local evidence - More commonly, the absence of negative incidents or outcomes meant PAs were simply regarded as performing in line with expectations - Evaluating the performance of a specific professional group raised concerns, as focusing on individuals or a smaller group felt discriminatory | **Not to my knowledge. I mean, there's been, what I would say, internal discussions with consultants saying, "Well, this person's really struggling. You need to work..." But no real evaluation. I don't think there has been any real evaluation. The three of us who do the mentoring for each of the PAs, there was no appraisal system for them. So I designed an appraisal that I used based on the GP appraisal system, which I've used with my PA, and was then used by the other PAs. But we fill these things in and sent them to the clinical lead for the unit. There was never any feedback…No point in you doing an appraisal if nobody reads it, and that's what I felt about. And it was nobody's fault because the clinical leads of the unit have got masses of responsibility and multiple emails. But actually, if you're going to put PAs into the hospital, you need somebody who's got the time and the capacity to really lead that process, then that should include proper evaluation of their role. - HA – TA 07 Clinician**  If it's working here, so we don't really, we don't have to prove it, if you know what I mean, because it's a role that's accepted at [Hospital C] and it's effective. - HC – CM 01 Manager  I haven't got any evidence that they're not delivering safe patient care. We haven't got any excessive of incidents related to PAs. Any concerns have been anecdotal but not substantiated. I have no evidence that, you know, PAs are any more likely than any other individual member of the team. – HB – CM 06 Clinician manager  There's no proof of safety, but there is proof of patient harm….Well, that takes several forms. One of them largely by its nature, is individually reported incidents or concerns shared by people, departments, or patients. Maybe that's because there hasn't been that research into how these PAs are being utilised, which would show evidence of safety. So where there has been evidence of harm is both through work that the BMA has done through surveys of doctors expressing concerns. – HD OT 01 – Resident doctors  So we did evaluate the…when we had the new group of PAs in, it's the pilot in medicine, we did evaluate that. And that was a business evaluation really, what does this business case meant, the KPIs... The main thing was a reduction in additional session spend for doctors because of the opening of overflow spaces, winter wards, et cetera. And an impact on flow, although it's very difficult to trace that impact on flow back to just the PAs. – HE – CM 01 Clinician manager  Not so much audit. Good question whether that should be done or not. It feels slightly wrong just to audit one workforce to assess their work and their competencies, and that probably feels like quite a discriminatory way to do that. So what we do is we just individually sign off their competencies, the same way that we would do if a junior doctor. But I guess if we wanted to break down some of the barriers, we could do an audit of their effectiveness, which hasn't been done within this organisation though. – HE – DM 01 Manager | - Across sites, participants generally described positive experiences with PAs, particularly in supporting team continuity and building relationships when junior team members rotated | - In some organisations (e.g. HA and HE), audits or appraisals of PAs had been undertaken, but these were not widely known among the participants |
| **Meso - Organisational leaders’ or champions’ perception and understanding** | - Organisational leaders or champions did not always keep up with macro-level issues affecting PAs, and felt they are constantly evolving - At the senior chief executive level, there were examples of leaders who understood the PA workforce and provided support, particularly in the recent climate - Some divisional management often seemed less aware of PA roles and their development across units - Leaders often held perspectives that differed from frontline team members, sometimes described as “utopian” - Some frontline team members considered that managers do not fully understand PAs’ roles and capabilities - At the clinical team level, team lead and consultants were described as the main champions of PAs, who played crucial roles in helping PAs integrate into teams | I think the resident doctors are frustrated. I think that they think that these guys are getting the easier ride that they would want, while they're staffed with the responsibility. Amongst the consultant grade on the other hand, especially those that have managerial roles and those senior doctors in managerial roles, of course they're given a different perspective...And I knew that amongst our executive, there are a lot of people that are very in favour of physician associates, critical care practitioners and so on. And they have like a very utopian vision of a multi-skilled workforce. But what they have in conversations with them, because my regular anaesthetist is one such person, when you speak with them, there is never a clear definition of where the PA role ends, for example, and where the SHO role starts, or the registrar role starts. – HA – TB 11 Clinician  I don't work with PAs. As far as I know, we have no physician associates working in our division. And I'm very careful of saying, if they are, then I'm not aware of them, nor would I necessarily have to be...I know we're with ACPs, I know we're with nurse consultants and I cannot recall a discussion or a paper about physician associates. So I would be surprised to assess one in my division. – HA - DM - 03 Nursing manager  **Our clinical director is also our undergrad lead as well, supports them. And I think it really is dependent on what your clinical director feels about them, and then how you embed them into an area. And I truly believe that. So there has been certain challenges with some areas definitely, but it keeps coming back to those two things about prescribing. – HB – CM 09 Manager**  So that's a challenge, and I think there's a challenge where hospital management in designing rotas doesn't understand the role of PAs necessarily, where actually they sometimes consider, or certainly that's happened where rota managers, nonclinical management, thinks that PAs and junior middle grade doctors or FY1s are interchangeable. When actually they are two discreet groups of people with separate responsibilities and skillsets, and actually also abilities in terms of prescribing and the legal restrictions around doing AWI, Adult With Incapacity Act certificates or detention certificates, short-term retention certificates in psychiatry, or in the emergency department or anywhere like that. That cannot be done by a PA. Some of these legal frameworks also cannot be done by PAs. So to put a PA in that position actually isn't feasible, but they still use them to stuff the rota or consider them as rota fodder. – HD OT 01 – Resident doctors  We did get a meeting with one of the senior leads of the hospital. She did give us her time and she seemed very pro-PA and very supportive of us. Have I seen much change? Probably not. But I think is that being limited by funding probably…I'd say this crisis point, of this horrible, lots of media, and hate was coming out, and people were feeling very concerned, and there was episodes of bullying within the trust...then we were able to, once with the PAs here, was able to message her, reach out and we did all meet. They probably would be able to have get better, to have more regular meetings with that. And obviously maybe more regular meetings, whether the whole PA cohort. – HE – OT 02 PA | - Although we did not interview all senior management, most organisations had at least one senior medical leader at the trust level who was aware and responsible for PA workforce issues - Champions, especially clinicians at the unit level, played a key role in integrating PA roles | - There were varied levels of engagement with macro-level issues for leaders and champions, some actively followed and commented on national debates, whereas others were less proactive and took more of a “wait and see” approach - Concerns over managers’ understanding of PA roles only occurs in one organisation |
| **Meso - Views of other departments and teams** | - Limited communications within organisations about PA roles and deployment, many staff were unsure where PAs were located in organisations - In some organisations, working with supportive units such as radiology and pharmacy has been more challenging, as well as referring to other specialties, impeding PAs’ clinical activities - Rotating and bank staff were occasionally being described as challenging to work with, as they often lacked understanding of PA roles | I first heard about the PA role at [Hospital A] when I was bleeped by one. And when I found out about it, I didn't actually know the person who bleeped me was a PA and I was slightly confused by somebody I thought was a doctor who was presenting the case in quite a different way. So it took a little bit more effort to get information and eventually when I saw the note that was written on the patient later on, I then realised it was a PA and probably was asking the wrong questions of that poor person on the phone. – HA – TA 05 Resident doctor  I think probably the difficulty lies with maybe other specialties who don't know you quite as well and probably have preconceived ideas probably too... I feel sometimes you do get a bit of reluctance or hesitation when accepting referrals from us. "Have they been seen by a doctor?".. regardless of what specialty they work in is just certain. There's certain individuals who I feel are reluctant to accept your views or referrals essentially. – HD TA 04 - PA  **There's specialties who don't have any PAs. And you still come across people who haven't worked with a PA, so they don't really know about whether they can relay this through to you or do they need to ask for an SHO or a registrar. So sometimes you get a call and they say, can I speak to your registrar? …But normally you just explain how it works and they understand. – HE – TB 02 PA** | - A common issue was rotating and bank staff had limited awareness of the PA role | - In some organisations (e.g. HC), radiology departments had stopped PAs requests for non-ionising radiation, which created challenges for PAs’ clinical work - More mentions about referrals in HD |
| **Meso - APP representation in organisations** | - Some organisations have lead PA roles at organisational level or departmental level, which played an important role in supporting role integration, increasing awareness, and representing PAs at senior level - In the current climate, it has been challenging to further promote and showcase PAs’ roles and activities - PA roles have increasingly become a topic of discussion in some organisations’ BMA local negotiating committee meetings - In some organisations, PAs felt they were not always well connected across different units | I think there has been quite a lot of negative aspects on like Twitter or social media and stuff. I've not personally seen a lot of these but I know the PAs were quite disheartened by a lot of it really, which is a shame because obviously we do like to promote all sessions that we do, it's nice to like showcase all the work that's happening and they don't want to put anything on social media in case there's any negative comments on that, which is a shame really because they are doing some amazing work and it would be nice to showcase, but it's made them not want to do anything with social media… when we had the showcase day, I asked if I could put some pictures on social media, and they asked me if I wouldn't, obviously, because of all the backlash and it was just making them feel really, really upset about it. – HB – CM 01 Other  The main conversation really is with the BMA and the, our local negotiating committee with the BMA because of the campaign that's being fought. So there are a, not many, but there are a number of people who are absolutely not recognised to PAs…being unreasonable in terms of individual PA behaviours towards PAs. – HB – CM 06 Clinician manager  And then they decided that they needed a lead for the PAs…So whether it's touching base with people, whether it's having meetings with people, whether it's organising the teaching schedule, whether it's when we come around to doing the self-roster, organising all of that and nitpicking the holes and getting that pulled through management to make sure they're agreed. – HD TA 02 - PA  I still think PAs work in silos, and we're not that good at all coming together to talk about these sort of things. I think there could be better support, but it's hard to say. I know that at least the surgical PAs, most of them, they work in groups anyways, so there's like three or four of them, and the medical ones a little bit as well…I would really like that to be more of a community, more of a like meet up. We keep talking about doing a social, but no one organises it. There's like 50 PAs on the chat, but you only ever hear from like 10 people. So I don't know what it is that needs to be done to get everyone together…I love working with my team anyway, and I think that's the case for everyone. They've kind of already found their little homes. But if that's not the case for someone who's working on their own, then that's rubbish. I definitely think there should be more PA to meet, togetherness. – HE – OT 01 PA | - Across sites, there was some awareness of local PA roles among senior managementPA themselves felt the shared concern of representing themselves in the current climate | - Two organisations (HC and HE) had a designated lead PA at organisational level (linked to having larger number of PAs), which supported integration, organisation and communication of training opportunities; for HD this was at the unit level; HB previously had a lead PA, while HA did not - PA issues were raised at LNC in most organisations, but in HC they were not reported as a discussion point |
| **Micro - APP individual background and attribute** | - PAs came into hospitals from a range of academic and professional backgrounds, including science, nursing and pharmacy - For some, prior familiarity with the hospital systems and settings helped them integrate more smoothly - However, varied backgrounds sometimes made it difficult for clinical team members to determine what PAs could do and how to assign tasks to them - Personal attributes were seen as highly influential, some PAs were seen as younger, enthusiastic, dynamic and communicative | **I've found them highly variable, where an F1 normally has a predictable level of practice. I've found that the PAs are quite different in how they operate, and it takes a little bit of time to then get used to working with them, so that you know what they're capable of, as opposed to one of their colleagues who I mean have the same qualification. I don't know if they've got the same training. I don't know if the training programmes are standardised or not standardised yet, I'm not too sure about that, but I have found their competencies quite varied. – HA – TA 03 Resident doctor**  When I started they were here, we had another PA, he was not doing very well and he was like in then after that a lot of discussion and he left the Trust. So these three are very good. But even doctors you can have good doctor, not confident enough, not competent and so like that, he was very slow and he was asking questions all the time… I think it's with him individually because he's very slow and processing is very slow. it's not related to PA role…- HC – TA 06 Clinician  I think within our integration of our PAs has been pretty well done actually. But I do wonder whether it might be personality specific as well. We've got a great bunch that are happy with, well, I shouldn't say that, but it feels as if they're happy in the roles that they're in at the moment and will do it very well. – HD TB 01 Clinician manager  They have a different work ethic, in my opinion, the PAs. The work ethic is closer to that of a junior doctor. The nurses, when they get into the specialist areas have got different…they clock in, clock out, very militant with all the timings. Whereas the physician associates, if they need to, they stay late, they do extra, they go above and beyond. Not that the nurse specialists don't, but because the PAs are younger generally, and they're really excited about doing the work, and they're more accepting of working in and around a busy [specialty] unit. – HE – TB 03 Clinician manager | - Personal attributes were seen as highly influential, with recognition that PAs bring diverse backgrounds and characteristics therefore need to be treated individually | - Cases of negative experiences with PAs such as in HA and HC were attributed to individual personality rather than the role |
| **Micro - APP individual skills and expertise** | - PAs’ competency levels varied by individual, some were trusted with greater responsibilities, but again this cause concern for clinical team members - Most PAs were considered strong in administrative tasks, knowledgeable about local processes and guidelines, and their longevity in clinical teams contributed to such expertise, despite others still doubt their level of competence | When we get calls from PAs we worry because we know that there'll be probably something missing in the history and that something won't be complete. And it's not through, I don't want to use the word incompetence 'cause it is, there are competencies that these people don't have. So it can't be done by that role, is my view on it. And they get very stressed because they also know that they can't. And sometimes what you hear on the phone, in the beginning I thought they were being a bit rude, but actually when I saw what they were dealing with, then I realised that, I think that they're just worried and need to be better supported in that. – HA – TA 05 Resident doctor  I really, really liked that (PAs) had been working in that [specialty] department for a few years now. And so they kind of, when I asked about, “What's the usual follow-up regime for this person, what do we usually prescribe for pain relief or anticoagulated medications?” They knew these protocols and these guidelines pretty set and well. Honestly, just because of how busy that department was and how many wards and outlier patients we looked after, I think the prescribing thing was just the biggest thing for me, … they helped relieve the administrative load, but then they were adding more prescribing load, but I did like that they were there to help with induction into that department and they were like a good source of knowledge for what the department usually does, protocol and guideline wise. – HB – TA 01 Resident doctor  **The very thing that their communication skills are very good in terms of communicating with their colleagues, doctors, nurses. The other thing, they communicate very well with patients, their depth of knowledge when it comes to the acute medical condition is surprising, like it's not what I expected…. They've been amazing so far actually. They're very helpful whenever you ask for anything because they've been in the Trust for a very long time, for a long time relatively -- relative to other trainee who rotate every few months. So, they know the local policies, they know all the guidelines. – HC – TA 08 Resident doctor**  I find that the two PAs know the patients really well, know how the ward works, know the nursing staff really well. So they're that kind of a really good bridge and they've got that continuity. So in terms of actual knowledge of patients, they're very, very good and very good at kind of understanding the patient journey and understanding what the plan is. So I find that really helpful. As a reg who's kind of also only occasionally dipping into a ward and then having to do a ward round and come up with plans for all these patients that I maybe don't know as well, it's really helpful that they do. So I often actually find it easier doing the ward round with them helping, with them kind of assisting than with one of the junior doctors. – HC – TB 03 Resident doctor  The argument would be that they don't have the depth of understanding or the breadth, but if they're always on the ward round and they're always on the ward with a junior doctor or somebody around them, and they're within their field of comfort, assessing an acute presentation, assessing blood pressure, assessing shock, putting lines in and things, they're quite efficient at that. But if you're on the same board or the same team, and you've worked there for five years, you've pretty much seen everything that comes in, and then they become more expert in their field. – HD TB 03 - Clinician | - Across organisations, PAs were consistently praised for their expertise around organisational structure and policy - PAs who stayed longer in post developed more specialised skills and expertise over time | - The types of specialist expertise PAs demonstrated were very linked to the characteristics of the specialty they work in, which is also linked to career development |
| **Micro - Relationships and negotiations with clinical team members and peers** | - Relationship with trainee doctors and nurses varied. Some found PAs very helpful, while others expressed tension when PAs were perceived as being treated preferentially (e.g. linked to clinical training resources, work pattern and also broadly salary banding) - Trainee sometimes expressed frustration and perceived them creating extra work. This was compounded by that many trainees had little prior knowledge or introduction of what PAs are, leading to confusion and resentment - PAs often had strong relationship with consultants and longer-standing clinical team members, as they had longer periods of working together, and this at times created challenges for junior rotating members - PA themselves often had to carefully manage and negotiate these relationships, and build relationship over many years, at times the restriction and limitation felt demoralising as PAs starting to think they are creating extra workload. Peer support from other PAs was described as particularly important in navigating tensions and supporting integration | That was the first time I'd heard about physician associates. They were in the unit when I arrived so they were already embedded in the unit and I've never worked with physician associates before, didn't really know what the role was about, what they could do, what they couldn't do, etcetera. So I've kind of just learnt while being on the job and working alongside them…Just by talking to them and working with them and seeing how they operate, what job roles they fulfil, and yeah, I didn't have an induction, so it was just by learning on the job. – HA – TA 03 Resident doctor  We do an induction morning. So the first day that the doctors rotate onto the ward, sorry, we have a meeting with all of the consultants, all of the new residents, and then myself and the other PA attend. And we usually just have a little chat after the consultants have said their piece, just introduce ourselves, explain our role within the department, and that we are there to help them as best as we can, but the limitations are no prescriptions and we can't request these scans. And I think just laying that out straight away so they know who we are, putting names to a face, and then when they see you on the ward, they're a lot more accepting of those issues, hindrances, rather than it just being sprung on them on their first day. So I think that it’s worked really well. I've been here for three rotations of junior doctors now, and that's worked every single time. It's worked really, really well. So I definitely think if other departments or trust would to probably do that, I think it could work quite well. – HC – TB 04 PA  I think other clinicians are a problem and management. They don't know what their limitations are. So I often have to say, "She is never independent. She is always under our guidance. She's highly talented, but she's not a registrar who can then do independent practice, or an ANP. She's not that." It's difficult…because she's supervised by doctors, but paid in a nursing funding, so that creates a lot of confusion as to, "Well, how can she be a Band 7 and not do things independently? – HC – OT 13 Clinician  Sometimes you do get like new junior doctors that have never worked with a PA before turn up and be like, oh I'm not sure about this. Like our last set I was doing, I think I was on nights or something and somebody asked me to like look at an ECG or someone asked, they asked one of the new FYs to look at an ECG and I said, oh I'm logged on if you want, I'll look at it. And they came and are you, are you OK? Are you happy to do that? Do you know how to look at an ECG? And I was like, it's been 12 years, I hope so, and they was like oh right, OK, that's fine, and then within a few days they're like asking you questions because they've realised that, you know, even just from the fact that you know how the systems work. even generally when they turn up and they're a bit unsure, within a few days they're like, they're usually like no, this is probably OK. – HD TA 03 - PA  PA don't have to rotate and they don't have to move. So they don't have to move from hospital to hospital. They've got this, some people would consider a cushion number, because they're there in the same place. They don't have to move. They're working 9:00 to 5:00. They're not doing weekends, they're not doing nights, and they're set, thank you very much, and they're doing things that I want to do. That seems to be the professional jealousy aspect to it in some places, that I think has started some of the antagonism as maybe simplistic. – HD TB 03 - Clinician  **It was challenging at first, being the first one. A lot of people hadn't heard of the role. If they had heard of the role, they didn't really know what I could or couldn't do, so there was a lot of education and learning on both sides, really. Me telling them what my training was, what I was allowed to do, not allowed to do, and also them thinking how they could fit me into their day-to-day running services. So it took a good few months, I'd say, just to find my feet and also just work out where I can fit in. – HE – TA 02 PA** | - Clinical team members’ views about PAs were often polarised, both among junior and senior medical and nursing staff | - Team dynamics varied depending on the number of PAs when compared to medical trainee. In some units, the number of PAs is significantly larger than medical trainee - Induction practices also differed, as in some organisations new clinical team members received structured introductions, and in others they were treated as part of the norm with no special induction |
| **Micro - Autonomy and relationships with supervisors** | - PAs often have both clinical and educational supervisors, though the arrangement varied widely - Clinical supervision was frequently mentioned, either directly or indirectly, and differed by specialty settings. For example, supervision arrangement was clearer in inpatient wards, while other areas it was less clear to the wider team - PAs reported that approaches and relationship with supervisors often evolve through time, and similarly supervisors reported that their approaches to supervision is linked to individual PAs’ competency and attribute - The supervision structure was not always clear to all other clinical members, junior team members often expressed concerns when supervising consultants were not present and when PAs asked them to order prescription | **In the different environments, she would be working in clinic where there would be a consultant alongside. In [specialty X] she would be working in clinic with a consultant alongside, in the assessment unit she'd be working as part of a team with a registrar and consultant, and in theatre she would be assisting a consultant or registrar so she was supervised….Not always in the room supervision, but indirectly supervised. – HB – DM 02 Clinician manager**  We have quite a bit of autonomy here, but yes, we are always supervised in terms of there's senior registrar and consultant support, but they don't check every single thing I do. They don't review all my patients.…And that was right from the get-go and I almost didn't feel comfortable with how much they were trusting me because I felt myself, I wasn't at that level of competency. And it took years for me to be comfortable with knowing my own limitations, if that makes sense. I wanted people to check what I was doing a bit more for the first couple of years. – HD TA 06 - PA | - PAs generally reported that their supervision and autonomy evolved over time - Other clinical team members recognised that PAs had supervision, but rotating clinical team members were less clear where the responsibilities lay when it comes to ordering prescription | - Concerns about supervision in acute emergency settings were commonly voiced by other specialties, whereas those in this specialty generally reported that the supervision was appropriate (e.g., HA TA and HC TA) |
| **Micro – Patient perception or preference** | - PAs reported that they explain their role clearly and deliberately, however this does not necessarily mean patients understand what PAs are or do, but this also apply to other roles - Some patients base their preference and experience more on the personality and exposure of the individuals rather than the profession - There are cases for patients to be seen by other team members such as doctors, but less common in hospital care especially urgent care | I think it's hard to say. Like I think definitely PAs introducing themselves and just I think stating their scope as well, because (patients are) not familiar with all these different terms. Even if you introduce yourself as a physician associate, they don't understand that that means you're not a prescriber or you don't do certain things, which I think would maybe affect how they request for different things. But to be honest, like I think especially with the inpatient ward at [Hospital B], all they're really concerned about is whether they get seen by a consultant or a senior registrar. And the difference in care, if I clinically review them, if they're acutely unwell or if the PA does it, to be honest, I don't think they see that difference…I think when they're wanting to hear about their management plan, they would rather hear it from a senior professional anyways. So the difference between me (foundation year doctor) and a PA is not super big for them. – HB – TA 01 – Clinician  No one has ever... There's maybe been a couple of (patients) who've said, "I want to see a doctor." But I always introduce myself as a PA. I specifically tell them I'm not a doctor, I work alongside the doctors. And most people are like, "Yeah, that's fine." They're not really that fussed… most of the time, if people are really unwell, they're not so much in a position to be requesting who they're seen by. – HD TA 04 - PA  **I have had patients call up through our [subspecialty] co-ordinator when I've been away, for example, asking can they delay their treatment until you come back because they want you to do it. Presumably that's just a they know me so they say I want my injections done by the same person each time as opposed to oh, you're a Physician Associate rather than a Nurse Specialist, which is sort of what that was. I've had that quite a few times in the past but I think that is just because they know me as a person rather than because I'm a Physician Associate. - HE – TA 03 PA**  We've had a couple of examples of (patients) who've said no. I think we've only had two examples, people who've said no and would only see a doctor. And that's made it very difficult to get treatments to them because the treatments are timed, and we just don't have enough [specialist] to do that. But also that the [specialist] aren't the specialists on moving medicines, on reviewing medicines, and so they needed support from me and the physician associate to do that task, because it's not something they normally do. So you need to see the person who does it the most, who's the most competent at that job, and not necessarily…– HE – TA 05 Other | - Poor or limited patient understanding of the PA role is a consistent theme across organisations | - Patient preference varied depending on the clinical settings, for example in urgent care patients are less concerned with the role and mostly want timely care (such as HD TA) |
| **Retention or career development** | | | | |
| **Macro – Career recognition, structure and pathway** | - Broad recognition that the PA role lacks career progression, with many stuck at band 7 - For some PAs, the role is valued for its generalist and broad scope and able to switch specialties - In some cases, compounded the currently negative climate, this lack of clear career progression has led PAs to reduce their FTE or even leave the profession as they felt there was no future | So talking generally about the PA role, in essence it was a great idea with not a great deal of forward thinking in terms of practically no one wants to work an award five days a week for the rest of their life, and there’s no progression from that. The post was developed at Band 7 with no potential for that to develop into anything else, because there's no prospect of career progression currently without you either going back to medical school, or going to medical school and taking that route or going into something else medical. So I think in essence it's not a terrible idea, but it's just not particularly being planned out in terms of longevity very well to support the PA workforce. – HB – TA 06 Other  Interestingly in the last year, four people have gone to less than full-time…Some of that is the stress of the department…plus the stress of the PA stuff. Up until the last year, as much as it was annoying and stuff, there wasn't really a huge impact in what we did…now the PA stuff started first, people are struggling more. – HD TA 02 - PA  I'm also worried, because I know a lot of PAs are actively choosing to leave the profession because they…so my own PA, like I said, is like my right hand woman and she is amazing and provides…every single patient who comes to me, who's already seen her, tells me how wonderful she is. She absolutely adds value and she's great at what she does, but she's taking a sabbatical next year because she's not sure if this is going to be the future for her. Because partly she can't really see career progression in it. Particularly, not now, but partly because she's not sure she wants to keep fighting this fight, and that's really sad. – HE – CM 01 Clinician manager  Now decision making is where I'm going to do another year on [specialty X], or whether I'll go back to [specialty Y]. So it's still, I've really enjoyed them both, and different things are great about different parts...This is a good thing about the PA role, you can try something and if you like it, and you work it well, then you are allowed, you can stay there. And actually it's transient. – HE - OT 02 PA | - Recognition that there is little to no career progression is common across organisations | - In some organisations (e.g., HC and HE) there are lead PA roles at band 8 whereas in others this does not exist, possibly linked to the number of PAs in each setting |
| **Meso – Organisational culture** | - A supportive organisational culture is important to retain PA, especially in this current challenging climate | In actual [team A], they stay around for quite a bit. The only ones that would be have a massive turnover rate would be the juniors, but then that's because they bounce around the hospital, so they do varying different things. For PAs, in the 18 months that I have been here, we've had the same PAs, so there hasn't been a massive turnover forum, which I would say is probably a good thing….we obviously have a nice environment to work in, and you get to know your people, your working relationships comes a bit better if you've not got a massive turnover. – HC – TA 07 Nurse  Why do they stay? They would stay because they are valued. They would stay because they work within a team where their skillset fits very well within the team that is requiring them to be there. – HD CM 03 – Clinician manager  **But the PAs I've got now have all stayed, and so they're about four, five years, some of them for five years, maybe longer. So, the recruitment and retention is fine. They don't tend to churn so much, and the current crop are obviously. We look after them quite carefully and so hopefully that means we provide a good environment for them to work, and the environment on the outside is not that supportive, so less likely to go somewhere else. – HE – TA 04 Clinician manager**  (The lead) been reassuring to me, as have the wider team about, "We love working with you, [PARTICIPANT]. You've got a job here." I've spoken with other consultants about changes to my role, and they've always said, "Look, if you prove that what you're doing is safe and document it, then no one can tell you you can't do." You can change your scope of practice, essentially. – HE – OT 01 PA | - Good and supportive culture is consistently recognised as important across organisations | - Not all units deliberately emphasised organisational culture in their responses |
| **Meso – Organisational leaders’ attitude** | - Senior chief executive level leaders are generally less directly involved with the PAs’ career progression - Leaders and senior clinicians need to provide a clear vision and supportive to explore new areas of professional development - Many remain uncertain about the ceiling for PAs - Some clinical leaders fear criticism or negative media attention when they actively promote and advance the PA role into specialist role | **I think you all depended on the individual PA and the relationship between your supervising consultant and how the department views the future of PAs. So it's all very dependent on which department you are, who your supervisor, and so on. So if you have a consultant supervisor who's very active, promotes PAs, you're more likely to develop more advanced skills…I think if you're in a department where there's less supervision and there's less promoting consultants, or supervised consultants are not more forward going, I think your development will just be static, become quite static over the years. I think it's all dependent on the department you go in and the leadership of the department, and their direction and their vision. – HC – OT 02-08 TB 01 – PA focus group**  There's nothing systematic really. It's all down to individual directorates. I think it would very much require the physician associates to express to their specialty colleagues how they want to develop. So there's nobody going in and saying, "This is what we want you to develop into." But when they've expressed an interest, then it's very much been taken on board. But yeah, the [specialty] one is the main one where things have developed in a particular way…And that's been a battle because they've been constantly under scrutiny from, for example, the local press. We went through a year or two where we'd regularly get freedom of information requests, basically reading between the lines, looking to see where they could criticise us for how we use our physician associates. But we've always held the line that we've done everything in a proper way, with appropriate supervision and under appropriate guidance from regulatory bodies. But yeah, it does make people wary about developing them to do other things. – HC – DM 01 Clinician manager  Since I've been looking after them I've been conscious to really try and help them train and develop as much as possible, I'll probably get on to it later, so I tried to set up training and get them access to e-portfolios and to make sure that they're being able to log their competencies in the advent that hopefully at one point they were going to get registration and governance from an external body. And knowing that if it was the GMC or any other body that the first thing that they were going to ask for was evidence of competence and evidence of guidance and working within frameworks, etcetera, so that they could ensure safe practice. So I've really tried to set about doing that over the last 9 or 10 years and to invest in them, develop training and teaching programmes, allocate study leave and study leave money. – HD TA 05 - Clinician | - There is acknowledgement that the individual clinical team leaders or some senior clinicians need to take initiative in advancing PAs’ career development | - While most leaders held a generally positive view of PAs, there are some (e.g., HB TA) where they are uncertain about the future progression of the role |
| **Meso – Organisational policy and arrangement** | - Policy guidance such as appraisal and job planning processes for PAs are not always clearly defined or communicated, and at some organisations this is being developed - Getting on higher banding (e.g. 8 or 8a) is challenging and requires moving into a leadership role but not always feasible | There's just no structure around it really. What we tend to do on the wards is try and treat them in the same way as the JCFs …We try and involve them in all our teaching activities, but they don't have any kind of formal assessment process other than your box standards trust annual appraisal. It's also slightly difficult in that they've been lumped in with the nurses from a management and pay point of view because they're all on Agenda for Change, so that makes it a bit more difficult to integrate with the more medical side ways of working…We have somebody who's nominated to be the lead for PAs from a medical side of things. We have a medical and a PA lead who attempts to engage with them about education and development. We've also appointed one of our PAs to be the PA lead as well. – HC - DM 01 Clinician manager  **‘It turns out there doesn't seem to be any structured pay structure. Some are a six, some are a seven, some are an 8A depending on the trust, which is ridiculous. And the criteria to move between six, seven and eight is based on nursing models in which a PA doesn't fit. So to convince the trust that she should be paid more, in the end we absolutely failed because we couldn't tick all those boxes for an 8A despite the fact she was performing like a registrar, but always with constant supervision. So we were completely stuck. - HC – OT 13 Clinician**  At the moment, we are having discussions at the level of more senior management regarding appraisal. That's our main objective at the moment, to ensure that our colleagues are registered with the GMC and that we've got a proper appraisal system in place. We are also looking at what the supervisors for our colleagues who are physician associates need to be able to do in order to facilitate their appraisal. – HD CM 03 – Clinician manager | - Lack of clear policy around appraisal and progression is consistently mentioned across organisations | - In some organisations (e.g., HC and HE), lead PA roles exist at band 8 level, whereas in others they do not, possibly linked to the number of PA in each organisation |
| **Meso – Organisational strategy and planning** | - Some organisations allow PAs to move into managerial roles, but often there is only capacity for one lead PA role - PAs can develop into specialist roles depending on clinical team needs, such as pairing with consultants in specialist clinics, and this is very much departmental driven, with some units managing this better than others - When clinical teams employ PAs for very specific tasks, progression can become challenging as new PAs will need to be hired to cover the previous role | **So I think that can be a challenge, because unlike a resident doctor who had to progress to a registrar role and a consultant role. There isn't that natural progression for PAs. But as part of the appraisal process, we have a personal development plan. So I encourage [PA colleague] to join our [specialty association]…So I tried to encourage them to take on wider roles outside…I think in terms of actually changing the role, that's a challenge because that's why I employed them. I employed them to do the role in perpetuity, I hope. So I think changing what they do on a day-to-day basis, is a challenge, but I don't see that they seek that. I think they seek to have the opportunities that I hope we provided for [PA colleague], in the wider national role. – HA – TB 02 Clinician manager**  Initially they just supported ward work; so that would be documenting during the ward round, developing their clinical skills, carrying out all the jobs on the ward round essentially and that was it and I would say that was the case for certainly the first 12 months. Then over time, the three that we have, have picked up sub-specialisation roles; so one of them works more in a clinic with [specific condition], another one specifically with [specific procedure] and another one within [specific condition]. – HB – TA 06 Other  So I've worked with PA in another department about five years ago who again, was mostly ward-based in a very similar role to what I've described kind of our PAs here were doing before. I think occasionally did some clinic stuff and kind of some quality improvement stuff and I think he's very much stayed in that role, kind of we're now sort of five years down the line. I don't think much has changed for him. So again, I think that the career development seems to be very kind of departmental, kind of determined by the department, really. – HC – TB 03 Resident doctor  What we've done with the PAs who've been around for a while, is they've then going to do some specialty clinics. So that gives them their development, that gives them, they're doing a one-on-one with a consultant. So they have a trusted PA with a trusted consultant who's doing a set piece of work that they report back individually, or they do them in parallel rooms, so that if you've got PAs right for a while, you don't want them to get bored, they can do a set piece that trainees can come in and out of, but that every week that needs to be done and there's always going to be somebody to do it. – HD TB 03 – Clinician  I guess the only problem is we can't implement too many of those lead (PA) roles. Once somebody gets them, they tend to stay for quite a long time and then we lose some of the more junior ones to those roles which are in other organisations. There's just not that natural progression still for them… And I think there are two PAs that have been here from the very beginning, who now obviously hold those lead roles. And whilst they will spend a lot of time training their colleagues, they then leave, so that there is still an issue where we don't have the proper progression route for them. – HE – DM 01 Manager | - Management and specialist clinical roles are the most commonly considered career progression pathways for PAs | - Again progression opportunities varied depending on the size of the PA numbers and departmental characteristics (e.g., whether focusing on outpatient clinics or specific procedures) |
| **Meso – Resources and opportunities for continued employment and development** | - Access to resources and development opportunities is largely clinical team and leader / supervisor driven - While continued employment is generally supported, some organisations face financial challenges and PAs are said to be the most likely affected - Career development often involves attending in-house or external training, which can have financial implications - These opportunities contributed not only to career development but also to retention | In the department, we have regular education opportunities. Twice a week we have radiology meetings, where one of the radiology consultants comes and discusses scans. You can take scans. Any scans that you want to discuss, you can take to that meeting. And then they will also discuss any interesting scans that they've seen. So that helps with the developing your radiology interpretation… we have teaching on a Tuesday. So normally that's either the consultants or a junior presenting cases, presenting audits, presenting…I'm one of the mortality and morbidity leads with one of the other PAs, so that's where we do our meetings and review cases that need to be looked at in more detail. And then on Wednesday we have junior led teaching, so there's different procedural skills that are available as well, like ultrasound, cannulation, the LP ascitic drains, simulations. There's continuous teaching opportunities available multiple times a week. We also get to attend…so as physician associate specifically, we attend the regional teaching once a month. So we're allowed to leave the ward for a day to attend that, which is really beneficial.. – HC – TA 02 PA  But we are very lucky here that we have had such good support and education. We went from, when I first started, we got a two-hour monthly teaching afternoon that had not long been introduced. And we've gone from having two hours to an entire day once a month. And we get all kinds of teaching now from our own staff, but also from staff within the hospital and it makes such a big difference. I think we're being invested in and it does make a huge difference to how much we can give back to the department. And it keeps us here as well. It keeps us interested, because it can be very wearing being in the same place, doing the same thing day in, day out if it's not providing any challenge or satisfaction. And actually we get all of that. – HD TA 06 - PA  We are cutting, we're losing 83 beds for medicine. So there's something very contextual here, but if we have an optimally functioning NHS that's maxed out on its efficiencies, that is strategic about its workforce, that has the right models of care in place, absolutely, your PAs are an asset. Because there's only so much work that you can give to the doctors that you have in the organisation. But if we're not in that environment, if we're in the challenges we're in now, unfortunately I think the PA workforce will be one of the first victims to fall with that. – HE CM 01 Clinician manager | - Career development typically centres around training courses, in house opportunities or taking on improvement or managerial roles | - Variation in the extent which PAs can access funding and resources, whether the resource is available to only medical team members or all staff - In some trusts (e.g. HB, HC, HE) financial challenges impacted PAs’ job security as well as access to financial resources for training - As career development is supervisor driven, some units have enabled PAs to take on national roles, and in one unit a PA progressed into a highly specialised role post training |
| **Micro – APP individual background** | - Many PAs are relatively young in their careers and see the role as requiring progression opportunities, as opposed to nurses advancing into ACP roles relatively late in their career - In some cases, individuals with prior professional backgrounds (e.g. nursing) have chosen to return to their original roles because of role limitation | I think as with many things, there's a bit of a gender split society. Is still, there are still some very strong gender traditions. I think a lot of PAs are still female. And when, for all of us, all women in these sorts of workplaces, there are easier alternatives sometimes to career progression. So if your career is not going quite as fast as you might want, there is a spillway, isn’t there, for women? We can go off and have our families or whatever. The men typically are a little bit more, or the younger women are a bit more, “Hey, this isn't what I projected. I'm off.”. – HA - TB 11 Clinician  I think progression wise, it's, at the moment it's very, very limited, which didn't really factor in when I was doing my training, but now I'm in the role, it’s become more, not of an issue, it's become more of a focus. I think that if we are to keep the role in the UK, which I think they need to, but I think they need to look at somehow identifying a way for progression or just some form of progression, because if you top out of Band 7 at the age of 25, 30, there's no further progression for you to go and do extra additional training or more senior roles. It limits your scope a little bit. – HC - TB 04 PA | - PAs generally being young was mentioned in several organisations, sometimes even raised as a concern for the whole profession | - Only a few organisations reported specific examples of PAs leaving to return to their original profession |
| **Micro – APP work experience and beliefs** | - Career development is largely initiated by individuals and supported at the clinical team level - Some PAs see themselves as generalists and prefer to move between specialties to maintain a ‘portfolio’ career, others prefer to deepen their expertise within a specialty, e.g., running specific clinics or procedures - Due to the absence of a clear pathway, many PAs observe the progression of medical trainees to inform their own development - A subset of PAs viewed the role as a 9-5 job because of personal interest or family - There are PAs who have left for medicine due to understanding of career ceiling and want to further developing in specific areas | There's nothing systematic really. It's all down to individual directorates. I think it would very much require the physician associates to express to their specialty colleagues how they want to develop. So there's nobody going in and saying, "This is what we want you to develop into." But when they've expressed an interest, then it's very much been taken on board. – HC – DM 01 Clinician manager  A lot of things depend on how the PAs are integrated into the department, and unfortunately, [specialty] didn't work the way that they wanted them to work. So when I got a job there, there was other two PAs. We worked alongside ANPs in the department, and the job was very repetitive. It was essentially elective wards, so people would come in for a day-case procedure and come home, and your main job was to make sure they're healthy enough for the procedure. You see them before, you see them after and make sure everything runs smoothly, and you discharge them. So the job itself was not stimulating enough. I thought I was getting really bored and I thought it was time to move on. And actually they couldn't retain PAs there and they stopped hiring. All of my team left pretty quickly. And I don't think they employed PAs for that specific ward, because I don't think PAs are meant to be in one specific ward. I think you start to lose your interest very quickly and you start to lose your other skills as well, because you're meant to be generalists throughout your career. And if you're really doing a very specific task, then it's easy to just lose all of your confidence in other things. So I moved on. – HE – TB 02 PA | - Developing by taking on specialist clinics or procedures is a common form of progression, often shaped by what PAs observe in the clinical team | - Switching specialties is relatively uncommon, most PAs interviewed had not moved, with only 1-2 cases reported in HB and HE. - Retraining in medicine is not a common option amongst the PAs we interviewed, only HC and HD |
| **Micro – Relationship with clinical team members** | - Consultants and supervisors played a key role in shaping PA experience and navigating team dynamics - Negative team relationships can lead to poor retention, but these are often described as issues with individual attributes rather than team challenges | Currently I'm enjoying working on the [Team B] ward and I do tend to stay on the ward for a longer period of time. I have gotten more involved with seeing some of the patients in follow-up clinics and in pre-admission clinics, so it's more just getting me more involved with the team and what I'm doing. – HA – TB 04 PA  But we did employ, recruit one PA which I think he did a 12-month probation and there was a lot of negative feeling around him as a person in that role. I think he had a nursing background and came in and he didn't kind of fulfil or didn't give any confidence at that time on the PA role. So he left. – HB – OT 02 Other  **I think it depends on what relationship you have with your team as well. Like I've been on the same ward for like two and a half years. The relationship with the nursing team, the physiotherapist, the wider MDT, as well as the doctors, and the people build their confidence in you and over time what you are able to do, what kind of discussions and decisions you are a part of. – HC – OT 02-08 TB 01 – PA focus group**  … I think this is really important is it's the way you approach things…if I recognise that the PA wanted or indeed the ANP wanted to do something but it actually wasn't going to enable them to progress in their area, OK, I would say, Doctor X needs this for their you know, for their training.…actually I think then you guys can watch…because you can really upset a team if you don't balance the opportunities and make sure that those opportunities have been divided up in the best way that can provide the optimum happiness. Not just happiness, optimum really progression of all of them. – HD CM 01 – Clinician manager | - All interviewed PAs reported generally positive relationships with their team members and indicated that this supported their intention to stay | - No clear differences across organisations, negative experiences were rare and mostly anecdotal |
